# Embryonic endothelial evolution towards first hematopoietic stem cells revealed by single-cell transcriptomic and functional analyses

**DOI:** 10.1038/s41422-020-0300-2

**Published:** 2020-03-20

**Authors:** Siyuan Hou, Zongcheng Li, Xiaona Zheng, Yun Gao, Ji Dong, Yanli Ni, Xiaobo Wang, Yunqiao Li, Xiaochen Ding, Zhilin Chang, Shuaili Li, Yuqiong Hu, Xiaoying Fan, Yu Hou, Lu Wen, Bing Liu, Fuchou Tang, Yu Lan

**Affiliations:** 1 Key Laboratory for Regenerative Medicine of Ministry of Education, Institute of Hematology, School of Medicine, Jinan University, Guangzhou, Guangdong 510632 China; 2 State Key Laboratory of Experimental Hematology, Fifth Medical Center of Chinese PLA General Hospital, Beijing, 100071 China; 3 Integrated Chinese and Western Medicine Postdoctoral Research Station, Jinan University, Guangzhou, Guangdong 510632 China; 4 State Key Laboratory of Proteomics, Academy of Military Medical Sciences, Academy of Military Sciences, Beijing, 100071 China; 5 Beijing Advanced Innovation Center for Genomics and Biomedical Institute for Pioneering Investigation via Convergence, College of Life Sciences, Peking University, Beijing, 100871 China; 6 Ministry of Education Key Laboratory of Cell Proliferation and Differentiation, Beijing, 100871 China; 7 Peking-Tsinghua Center for Life Sciences, Peking University, Beijing, 100871 China; 8 Guangzhou Regenerative Medicine and Health-Guangdong Laboratory (GRMH-GDL), Guangzhou, Guangdong 510530 China

**Keywords:** Haematopoietic stem cells, Developmental biology

## Abstract

Hematopoietic stem cells (HSCs) in adults are believed to be born from hemogenic endothelial cells (HECs) in mid-gestational embryos. Due to the rare and transient nature, the HSC-competent HECs have never been stringently identified and accurately captured, let alone their genuine vascular precursors. Here, we first used high-precision single-cell transcriptomics to unbiasedly examine the relevant EC populations at continuous developmental stages with intervals of 0.5 days from embryonic day (E) 9.5 to E11.0. As a consequence, we transcriptomically identified two molecularly different arterial EC populations and putative HSC-primed HECs, whose number peaked at E10.0 and sharply decreased thereafter, in the dorsal aorta of the aorta-gonad-mesonephros (AGM) region. Combining computational prediction and in vivo functional validation, we precisely captured HSC-competent HECs by the newly constructed Neurl3-EGFP reporter mouse model, and realized the enrichment further by a combination of surface markers (Procr^+^Kit^+^CD44^+^, PK44). Surprisingly, the endothelial-hematopoietic dual potential was rarely but reliably witnessed in the cultures of single HECs. Noteworthy, primitive vascular ECs from E8.0 experienced two-step fate choices to become HSC-primed HECs, namely an initial arterial fate choice followed by a hemogenic fate conversion. This finding resolves several previously observed contradictions. Taken together, comprehensive understanding of endothelial evolutions and molecular programs underlying HSC-primed HEC specification in vivo will facilitate future investigations directing HSC production in vitro.

## Introduction

The adult hematopoietic system, consisting mainly of hematopoietic stem cells (HSCs) and their multi-lineage progenies, is believed to be derived from hemogenic endothelial cells (HECs) in mid-gestational embryos.^1,2^ It is generally accepted that while still embedded in the endothelial layer and presenting endothelial characteristics, HECs begin to express key hemogenic transcription factor (TF) Runx1 and have hemogenic potential.^3,4^ Different from hematopoietic progenitors, HECs lack the expression of hematopoietic surface markers, such as CD41 and CD45, which mark the population capable of generating hematopoietic progenies when directly tested in colony-forming unit assays.^3,5^ Hematopoietic stem and progenitor cells (HSPCs) are visualized to emerge from aortic endothelial cells (ECs) via a transient and dynamic process called endothelial-to-hematopoietic transition to form intra-aortic hematopoietic clusters (IAHCs).^6–10^ Being located within IAHCs or to the deeper sub-endothelial layers, pre-HSCs serve as the important cellular intermediates between HECs and HSCs, featured by their inducible repopulating capacity and priming with hematopoietic surface markers.^11–15^ The specification of HSC-primed HECs is the initial and one of the most pivotal steps for vascular ECs to choose HSC fate. However, as the precise identity of HSC-primed HECs is not clear, contradictory notions regarding whether primordial or arterial fated ECs are the direct origin of HSC-primed HECs are still under debate. It is proposed that definitive HECs and arterial ECs represent distinct lineages.^16,17^ Moreover, HSCs and arterial ECs are proposed to arise from distinct precursors, characterized by different Notch signaling strengths.^18^ Most recently, HSC-primed HECs have been transcriptionally identified in human embryos, which present an unambiguous arterial property, indicative of their arterial EC origin.^19^

In order to deeply investigate the cellular evolutions and molecular events underlying the specification of HSC-primed HECs and their subsequent commitment to HSPCs, it is necessary to efficiently isolate the HSC-primed HECs, which is proven to be difficult not only because the population is proposed to be small and transient, but also due to the technical challenges to determine their HSC competence.^20^ Considering that not only HSCs but also the transient definitive hematopoiesis during embryogenesis are derived from HECs, repopulating capacity is required for the functional evaluation of the HSC-primed HECs. Previous studies have reported the HSC competence of CD47^+^ but not CD47^−^ ECs in embryonic day (E) 10.5 aorta-gonad-mesonephros (AGM) region and both Dll4^+^ and Dll4^−^ ECs in E9.5 para-aortic splanchnopleura (P-Sp) region.^11,21^ Nevertheless, the enrichment of the above surface markers is far from efficient. Several transgenic reporter mouse models have been established by which HECs could be distinguished from non-HECs, including *Ly6a-GFP*, *Runx1* +*23GFP* (GFP transgenic reporter under the control of Runx1 +23 enhancer) and *Gfi1-Tomato*, and the usage of these reporters largely helps to delineate the process of endothelial-to-hematopoietic transition.^3,14,22–25^ Although expected to a certain extent, the HSC competence of the HECs labeled by these reporters has not been directly validated. Up to date, efficient isolation of the HSC-primed HEC population has not yet been achieved.

With the aim of delineating the molecular events underlying HSC emergence, several single-cell transcriptional profiling studies on HECs, IAHC cells, and HSPCs in the AGM region have been reported in recent years. Using either Runx1 +23GFP or Gfi1-Tomato as the marker of putative HECs, several defined cell populations were transcriptionally profiled by Fluidigm single-cell qPCR or single-cell RNA sequencing (scRNA-seq).^3,14,22^ Moreover, the cellular components of IAHCs were investigated at single-cell level by mechanically picking up single whole IAHCs in the aortas, showing cells with pre-HSC feature are predominantly involved.^14^ Interestingly, contradiction still exists regarding whether HECs and non-HECs are molecularly similar and to what extent the two populations are distinguishable.^14,22^ Since the enrichment efficiency or specificity of the above markers to define the HEC population might be not enough, an unsupervised screening of the embryonic endothelial pool within hematopoietic tissues is required for the precise recognition of HSC-primed HECs.

As compared to human embryos, mouse embryos serve as good models for the researches on the developmental events, especially the development of rare and transient cell populations such as HECs, due to the considerable and schedulable resources, the convenience in the in vivo functional evaluation, and most importantly, the feasibility of genetic manipulations. Here, we first used high-precision single-cell transcriptomics to unbiasedly examine all the EC populations spanning continuous developmental stages covering the presumed time points for the specification of HSC-primed HECs, transcriptomically identified them, and computationally screened for their candidate markers. Based on the consequently precise capture and isolation of the HSC-competent HECs using a combination of surface markers or newly constructed fluorescent reporter mice, we further decoded the cellular evolutions and molecular programs underlying the stepwise hemogenic fate settling from the initial primordial vascular ECs. A series of new findings, including the endothelial-hematopoietic dual potential of HECs and the multi-step fate choice for the specification of HSC-primed HECs, unprecedentedly enrich our understanding of HSC generation in vivo and should be extremely critical to inspire new approaches for stepwise HSC regeneration from pluripotent stem cells.^26^

## Results

### Transcriptomic identification of HECs in the AGM region

**Fig. 1:**
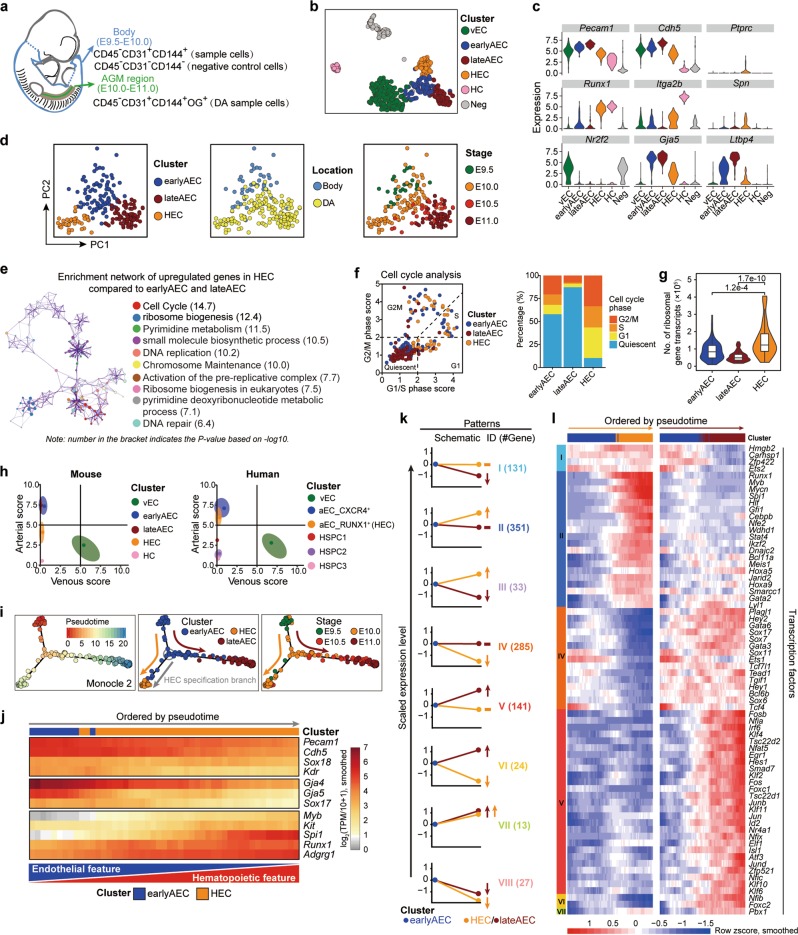
Transcriptomic identification and molecular characteristics of the HECs in the AGM region. **a** Schematic illustration of the strategies used for embryo dissection and cell preparation for the subsequent scRNA-seq. The involved body part and the AGM region is indicated as blue and green, respectively, with head, limb buds, heart, visceral bud, and umbilical and vitelline vessels outside the embryo proper excluded. **b** t-SNE plots with clusters mapped onto it. **c** Violin plots showing the expression levels of indicated genes in six clusters identified in the initial dataset. **d** PCA plots with three clusters (earlyAEC, lateAEC and HEC) (left), sampling locations (middle) and embryonic stages (right) mapped onto it. **e** Metascape network enrichment analysis with top 10 enriched terms exhibited on the right. Each cluster is represented by different colors and each enriched term is represented by a circle node. Number in the bracket indicates the *P* value based on −log10. **f** Classification of the indicated cells into quiescent phase and other cycling phases (G1, S and G2M) based on the average expression of G1/S and G2/M gene sets (left). Stacked bar chart showing the constitution of different cell cycle phases in the three corresponding clusters shown on the left (right). **g** Violin plot showing the number of transcripts for ribosome-related genes detected in each single cell of the indicated clusters. Wilcoxon Rank Sum test is employed to test the significance of difference and *P* values are indicated for the comparison. *P* < 0.05 is considered statistically significant. **h** Scatterplot showing the average arteriovenous scores of the cells in each cluster for mouse dataset in this paper (left) and human dataset from a published article^19^ (right), respectively. Main distribution ranges of arteriovenous scores in each cluster are also indicated as an oval shape. **i** Pseudotemporal ordering of the cells in three selected clusters inferred by monocle 2, with pseudotime (left), clusters (middle) and sampling stages (right) mapped to it. HEC specification and AEC maturation directions are indicated as orange and deep red arrows, respectively. **j** Heatmap showing the expression of the indicated genes (smoothed over 15 adjacent cells) with cells ordered along the pseudotime axis of HEC specification branch inferred by monocle 2. **k** Eight major expression patterns identified from the differentially expressed genes in HEC or lateAEC as compared to earlyAEC. Arrows showing the changes in HEC or lateAEC as compared to earlyAEC. The numbers of pattern genes are indicated on the right. **l** Heatmaps showing the relative expressions (smoothed over 20 adjacent cells) of the TFs belonging to the pattern genes with cells ordered along the pseudotime axis and genes ordered by patterns.

We first analyzed mouse embryos from E9.5, when the initial IAHC formation in the aorta occurs,^7^ to the stage of the appearance of HSCs at E11.0^27^ (Supplementary information, Fig. S1a). The embryo proper was isolated, and the vitelline and umbilical vessels outside the embryo proper as well as head, limb buds, heart, and visceral bud were excluded (Fig. 1a). To specifically capture the aortic luminal ECs in the AGM region, we performed microinjection of fluorescent dye Oregon green into the dorsal aortas of E10.0–E11.0 embryos as reported^12^ (Fig. 1a; Supplementary information, Fig. S1b). The sampled cells were purified by FACS as CD45^−^CD31^+^CD144^+^, which contained predominantly vascular ECs and CD41^+^ hematopoietic cells. Meanwhile, CD45^−^CD31^−^CD144^−^ non-EC cells in the body were used as negative controls (Fig. 1a). We used unique molecular identifier (UMI)-based scRNA-seq method to accurately measure the gene expression profiles within individual cells. In totally 662 sequenced single cells, 597 single-cell transcriptomes passed rigorous quality control. On average we detected 7035 genes (from 2266 to 10,843) and 636,418 transcripts (from 103,793 to 2,959,573) expressed in each individual cell (Supplementary information, Fig. S1c).

According to a graph-based clustering approach from Seurat software,^28^ all cells were separated into six clusters, including one negative (Neg) cluster composed of mainly non-EC negative control cells, and five sample clusters comprising almost all FACS-isolated sample cells (Fig. 1b; Supplementary information, Fig. S1d and Table S1). Featured by the obvious *Runx1* and *Itga2b* (encoding CD41) expression, the CD45^−^ hematopoietic cell (HC) cluster was distributed away from the other four vascular EC clusters that presented apparent arterial or venous characteristics (Fig. 1b, c). One venous EC (vEC) cluster was readily recognized by the exclusive expression of *Nr2f2* in all vascular EC populations (Fig. 1b, c). Two arterial EC clusters showed similar *Gja5* expression but different levels of *Ltbp4* expression.^29^ Together with their different sampling stages (mainly from E9.5–E10.0 and E10.5–E11.0, respectively), they were annotated as early arterial EC (earlyAEC) and late arterial EC (lateAEC), respectively (Fig. 1b, c; Supplementary information, Fig. S1d). The left one cluster basically met the criteria of the molecular definition of HEC, showing apparent *Runx1* expression upon endothelial properties, and was consequently named as HEC cluster (Fig. 1b, c). No batch effect was detected for different experiments (Supplementary information, Fig. S1e), indicative of the high quality and reproducibility of the scRNA-seq procedures in the present study. To more strictly define the HEC population, cells within the Neg cluster and those transcriptionally expressing *Ptprc* (encoding CD45) or *Spn* (encoding CD43) were excluded for the subsequent analysis (Supplementary information, Fig. S1f).

HEC and the other two AEC clusters were further focused on as they were either molecularly or spatiotemporally near to each other (Fig. 1b, d). To exclude the possibility that we failed to identify important populations relevant to hemogenic specification in earlyAEC cluster, which contributed evidently to the aortic inner layer of AGM region at E10.0 (Supplementary information, Fig. S1f), we performed forced clustering within the given cluster. *Runx1* (signature of hemogenic specification) was not significantly differentially expressed between the two sub-clusters, suggesting that no population with sign of hemogenic specification was missed by our clustering (Supplementary information, Fig. S1g). Moreover, very few genes were significantly differentially expressed in the sub-clusters of HEC by forced clustering, and none of them was related to hemogenic or hematopoietic features, indicative of the largely homogeneous property of the HEC cluster (Supplementary information, Fig. S1g). The HEC population was reduced promptly at E10.5, and became hardly detectable by E11.0 (Fig. 1d; Supplementary information, Fig. S1f). The highly expressed genes in HEC as compared to earlyAEC and lateAEC were mainly enriched in terms related to cell cycle and ribosome biogenesis (Fig. 1e; Supplementary information, Table S2). Cell cycle analysis demonstrated a remarkably activated cycling in HEC, in sharp contrast to the quiescent state by arterial EC maturation (Fig. 1f). On average, each cell in the HEC cluster expressed more mRNA molecules and ribosomal genes than either earlyAEC or lateAEC (Fig. 1g; Supplementary information, Fig. S1h), supportive of the globally up-regulated transcriptional and translational activity during hemogenic specification, which was in line with the finding in human embryo that translational initiation is more widely present in HSC-primed HECs than in arterial ECs.^19^ We further evaluated the arteriovenous scores of the populations we defined, and found similar results in mouse and human that HEC rather than hematopoietic populations manifested certain arterial features (Fig. 1h).

Trajectory analysis by Monocle 2 suggested that along the arterial maturation path from earlyAEC towards lateAEC, HEC was segregated out from earlyAEC at E9.5-E10.0 (Fig. 1i). The gradual up-regulation of hemogenic genes, including *Runx1* and *Spi1*, was accompanied by the gradual down-regulation of both endothelial and arterial genes along the HEC specification pseudotime, with the endothelial-hematopoietic dual feature of the HEC population presenting as a dynamic continuum (Fig. 1j). The finding was in line with a previous report about the reciprocal expression of Runx1 and Sox17 in HECs.^30^ To search for genes that would potentially contribute to the distinct fate choices of earlyAEC, those differentially expressed between earlyAEC and its downstream population HEC or lateAEC were picked up, and eight major patterns of expression variation were witnessed (Fig. 1k; Supplementary information, Fig. S1i and Table S3). Most of these genes showed altered expression along one unique specification path from earlyAEC (Pattern I, II IV, and V) (Fig. 1k; Supplementary information, Fig. S1i). Most TFs within these groups showed up-regulated expression along either HEC specification or arterial EC maturation (Fig. 1l; Supplementary information, Fig. S1j). Interestingly, the expressions of both *Hoxa5* and *Hoxa9* followed the same pattern as that of *Runx1*, although the formers were not exactly correlated with the latter (Fig. 1l; Supplementary information, Fig. S1j, k). The data suggested that the expression of genes should be orchestrated and precisely regulated for the subsequent cell fate choice from earlyAEC.

### Efficient capture of the HSC-competent and endothelial-hematopoietic dual-potent HECs in the AGM region

**Fig. 2:**
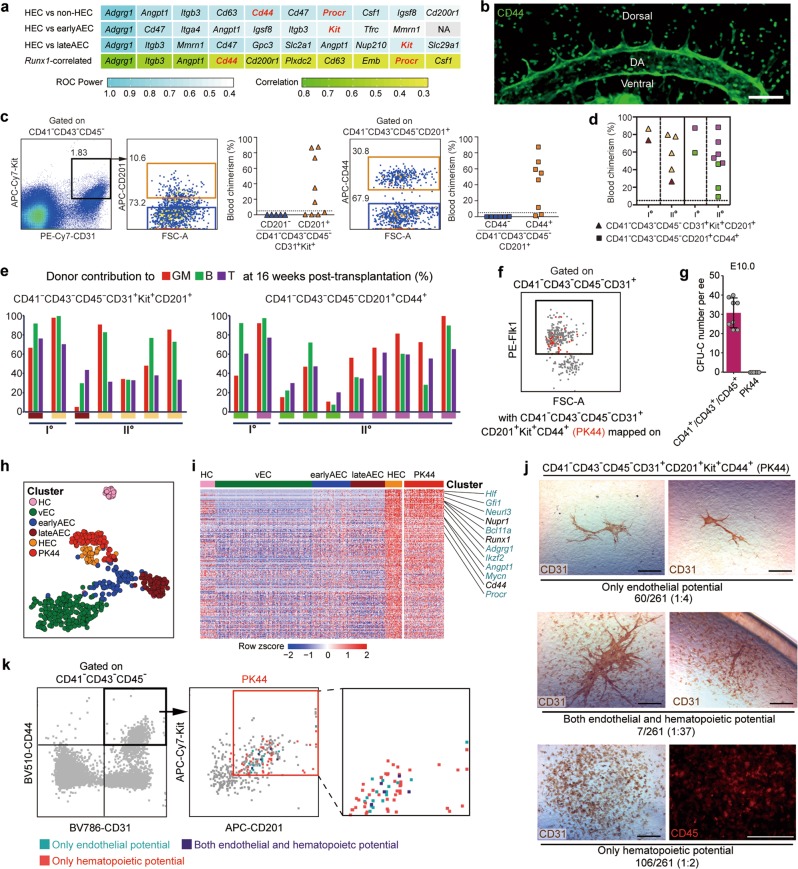
Efficient isolation of the HSC-competent and endothelial-hematopoietic dual-potent HECs before HSC emergence. **a** Gene lists of the top ten cell surface molecules significantly overrepresented in HEC as compared to the indicated cell populations (first 3 lines) and those positively correlated with *Runx1* within 4 EC clusters (vEC, earlyAEC, lateAEC and HEC, last line). Non-HEC, cells except for HEC within 4 EC clusters. Highlights in red font indicate the candidates used for further functional analysis. **b** Representative whole-mount staining of CD44 at E10.0 AGM region, showing CD44 is expressed in the whole endothelial layer of the dorsal aorta and roots of its proximal branches. DA, dorsal aorta; Scale bar, 100 μm. **c** Representative FACS plots for cell sorting of the E9.5-E10.0 caudal half for co-culture/transplantation assay and the donor chimerism at 16 weeks after transplantation of the derivatives of the indicated cell populations. **d** Blood chimerism of the primary (I^o^) and corresponding secondary (II^o^) recipients at 16 weeks post-transplantation. The primary recipients were transplanted with the derivatives of the indicated cells from the caudal half of E9.5-E10.0 embryos. The paired primary and corresponding secondary repopulated mice are shown as the same symbol and color. **e** Bars represent the percent donor contribution to the granulocytes/monocytes (GM, red), B lymphocytes (green), and T lymphocytes (purple) in the peripheral blood of the primary (I°) and secondary (II°) recipients at 16 weeks post-transplantation. The paired primary and corresponding secondary repopulated mice are shown as the same colors below. **f** FACS plot of Flk1 expression in the indicated population of E10.0 AGM region, with PK44 (CD41^−^CD43^−^CD45^−^CD31^+^CD201^+^Kit^+^CD44^+^) cells (red) mapped onto it. Box indicates the gate of Flk1^+^ cells. **g** Number of hematopoietic progenitors per embryo equivalent (ee) in the indicated populations derived from E10.0 caudal half measured by the methylcellulose colony forming unit-culture (CFU-C) assay. Data are means ± SD. Data are from 4 independent experiments. **h** t-SNE plot of the cells included in the filtered initial dataset and PK44 dataset, with clusters mapped on it. PK44, CD41^−^CD43^−^CD45^−^CD31^+^CD201^+^Kit^+^CD44^+^ population from E10.0 AGM region. **i** Heatmap showing the relative expressions of HEC feature genes, which are defined as those significantly highly expressed as compared to others including HC, vEC, earlyAEC and lateAEC, in the indicated cell populations. Selected HEC feature genes are shown on the right with pre-HSC signature genes marked as aquamarine. **j** Representative CD31 and CD45 immunostaining on the cultures of single PK44 cells from E10.0 AGM region, showing typical morphologies regarding distinct differentiation potentials. Cell frequencies of each kind of potential are also shown. Data are from 5 independent experiments with totally 15 embryos used. Scale bars, 400 μm. **k** Expression of Kit and CD201 in the index-sorted single PK44 cells with differentiation potential based on in vitro functional evaluation. Cells with different kinds of potentials are mapped onto the reference FACS plots (gray dots). Box in the middle plot indicates the gate for FACS sorting of PK44 cells in E10.0 AGM region and its enlarged view is shown on the right.

We next made an effort to identify surface marker combination to effectively enrich the HECs for functional evaluation (Supplementary information, Table S4). *Cd44*, *Procr* (coding CD201) and *Kit* were screened out by analysis of differentially expressed genes among populations and expression correlations with *Runx1* (Fig. 2a). We specifically focused on E10.0 in the following functional assays to keep consistent with the transcriptomic findings. Whole-mount immunostaining showed that in addition to the scattered blood cells throughout the tissue, the expression of CD44 was detected in the whole endothelial layer of dorsal aorta and very proximal part of its segmental branches (Fig. 2b), in line with a previous report about the arterial endothelial localization of CD44 in mouse embryos.^31^ Using the similar strategy as for pre-HSC identification,^11^ we found that only the derivatives of CD41^−^CD43^−^CD45^−^CD31^+^Kit^+^CD201^+^ rather than CD41^−^CD43^−^CD45^−^CD31^+^Kit^+^CD201^−^ population at E9.5–E10.0 could long-term (16 weeks) and multilineage reconstitute lethally irradiated adult recipients, although both populations generated hematopoietic clusters with different frequencies upon 7 days of culture on OP9-DL1 stromal cells (Fig. 2c–e; Supplementary information, Fig. S2a–c). Self-renewal capacity of the HSCs was further validated by secondary transplantation (Fig. 2d, e; Supplementary information, Fig. S2b). Within CD41^−^CD43^−^CD45^−^CD201^+^ population, induced HSC potential was exclusively detected in CD44^+^ subpopulation (Fig. 2c–e; Supplementary information, Fig. S2a–c). Thus, our data identified CD41^−^CD43^−^CD45^−^CD31^+^CD201^+^Kit^+^CD44^+^ (abbreviated as Procr^+^Kit^+^CD44^+^, PK44) population in E10.0 caudal half as the HSC-competent HECs.

As compared to other endothelial surface markers, Flk1 (encoded by *Kdr*) is known to be specifically localized within the vessel lumen layer, and very few and only the basal-most localized IAHC cells express Flk1.^7^ Here, almost all PK44 cells expressed Flk1 as shown by FACS analysis, indicative of their endothelial layer localization (Fig. 2f). Furthermore, the hematopoietic colony forming capacity in methylcellulose culture medium was absent in PK44 population, validating its identity as ECs rather than committed hematopoietic progenitors (Fig. 2g). To determine the transcriptomic identity of these HSC-competent HECs, we isolated PK44 cells derived from E10.0 AGM by flow cytometry and performed scRNA-seq with totally 96 of them (Supplementary information, Table S1). The sequenced PK44 cells were localized together with HEC by computational assignment (Fig. 2h), and showed a similar high expression of the HEC feature genes (Fig. 2i). The ubiquitous and obvious expression of several key hematopoietic TFs, including *Runx1*, *Spi1*, *Gfi1*, and *Myb*,^3,22^ in PK44 cells inferred the enrichment of hemogenic potential (Fig. 2i; Supplementary information, Fig. S2d). Therefore, immunophenotypically purified PK44 cells elegantly represented the transcriptomically defined HECs.

We next explored whether endothelial-hematopoietic dual potential could be detected in these HSC-competent HECs, since a transient intermediate state might be captured in the cell population experiencing fate choice. Firstly, we found that the Kit^+^CD201^+^ ECs in the body part of embryo proper at E9.5–E10.0 had a relatively higher endothelial tube-forming capacity as compared to the Kit^−^ or Kit^+^CD201^−^ endothelial populations (Supplementary information, Fig. S2e). Furthermore, CD44^+^ and CD44^−^ fractions within Kit^+^CD201^+^ ECs showed comparable endothelial tube-forming capacity whereas the generation of hematopoietic cells in the cultures was exclusively detected in the CD44^+^ ones under the endothelial-hematopoietic dual potential induction system (Supplementary information, Fig. S2f). The data suggested a largely maintained endothelial potential in the HECs. By single-cell in vitro induction, 40.6% (106/261) of PK44 cells gave rise to only hematopoietic progenies and 23.0% (60/261) only endothelial tubules. Remarkably, 2.7% (7/261) had both endothelial and hematopoietic potential (Fig. 2j; Supplementary information, Fig. S2f). All three kinds of potentials did not present an obviously biased distribution regarding Kit or CD201 expression level as indicated by index sorting analysis (Fig. 2k). Such low frequency of the dual potential should properly represent the intermediate cellular state in HECs along their specification path (Fig. 1j), with both endothelial and hematopoietic competencies being reflected by the asymmetric cell division under in vitro culture condition, which further emphasized the high efficiency of capturing such a dynamic functional population via unsupervised computational screening.

### Transcriptional and functional relationship between HECs and T1 pre-HSCs

**Fig. 3:**
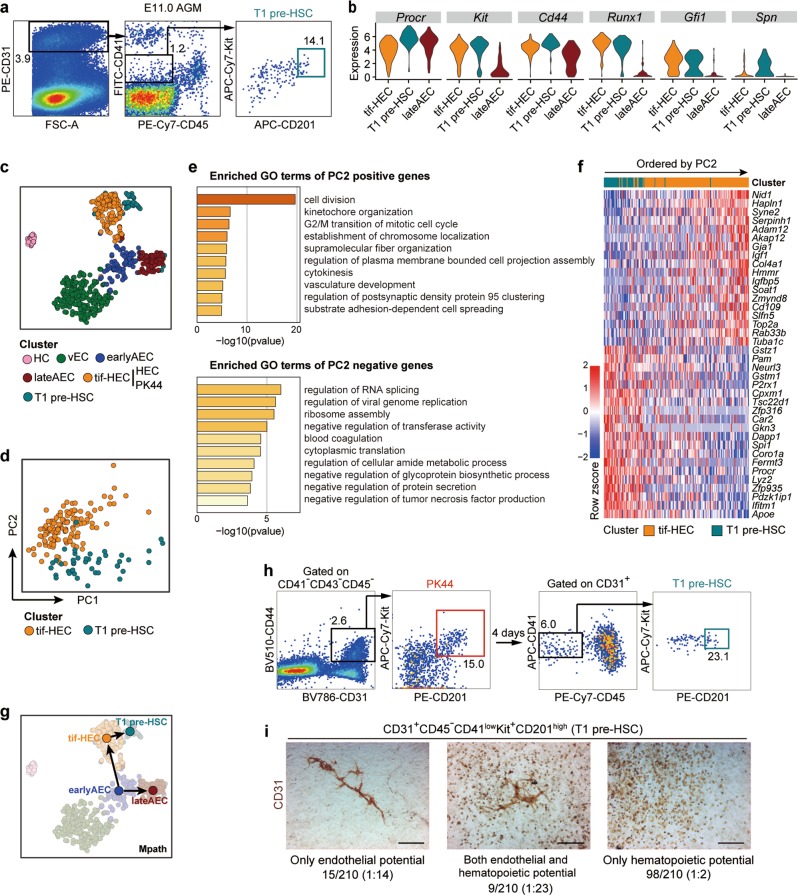
Relationship between HSC-primed HECs and T1 pre-HSCs. **a** Representative FACS plots for sorting of the T1 pre-HSCs (CD31^+^CD45^−^CD41^low^Kit^+^CD201^high^) from E11.0 AGM region of mouse embryos for the subsequent scRNA-seq. **b** Violin plots showing the expression levels of indicated genes in tif-HEC (including clusters HEC and PK44), T1 pre-HSC and lateAEC. **c** t-SNE plot of the cells included in the filtered initial dataset, PK44 dataset and T1 pre-HSC dataset, with clusters mapped on it. Clusters HEC and PK44 are combined as tif-HEC. **d** PCA plot of tif-HEC and T1 pre-HSC populations. **e**. Enriched terms of PC2-positive and -negative genes are shown, corresponding to the properties distinguishing tif-HEC and T1 pre-HSC, respectively. **f** Heatmap showing top 20 positive and negative genes of PC2. Genes are ordered by their contributions to PC2. **g** Trajectory of AEC clusters, tif-HEC and T1 pre-HSC inferred by Mpath. Arrows indicate the development directions predicted by sampling stages. **h** Representative FACS plots for sorting of the PK44 cells from E10.0 AGM region (left) and analysis of the immunophenotypic T1 pre-HSCs (right) after cultured in vitro for 4 days. **i** Representative CD31 immunostaining on the cultures of single T1 pre-HSCs from E11.0 AGM region, showing typical morphologies regarding distinct differentiation capacities. Cell frequencies of each type are also shown. Data are from 7 independent experiments with totally 89 embryos used. Scale bars, 400 μm.

Since the transcriptomically identified HECs and the sequenced immunophenotypically defined HSC-competent HECs (PK44) presented a largely similar molecular features (Fig. 2h, i; Supplementary information, Fig. S2d), we combined and designated them as transcriptomic and immunophenotypic and functional HEC (tif-HEC) for the subsequent analysis. The tif-HEC expressed a series of pre-HSC signature genes we previously identified,^11^ including *Hlf*, *Gfi1*, *Neurl3*, *Bcl11a*, *Adgrg1*, *Ikzf2*, *Angpt1*, *Mycn*, and *Procr*, suggestive of their HSC-related identity (Fig. 2i). We further performed scRNA-seq of 47 T1 pre-HSCs (CD31^+^CD45^−^CD41^low^Kit^+^CD201^high^) isolated from E11.0 AGM^11^ using the same sequencing strategy as other cells in the present study (Fig. 3a; Supplementary information, Table S1). As compared to tif-HEC, T1 pre-HSC expressed similar level of *Runx1* and *Gfi1* but obviously higher level of *Spn* (encoding CD43), validating its hematopoietic cell identity (Fig. 3b). The distribution of most T1 pre-HSCs was adjacent to tif-HEC via t-SNE visualization (Fig. 3c). Of note, principal component (PC) 2 by PCA analysis largely captured the transcriptomic differences between tif-HEC and T1 pre-HSC (Fig. 3d). The genes enriched in PC2-positive direction, where tif-HECs were mainly localized, were related to cell division, vascular development and cell spreading (Fig. 3e). Consistently, approximately 90% of cells in the HEC cluster were proliferative (Fig. 1f), whereas the constitution is only about half in the T1 pre-HSCs.^11^ Serving as the extracellular matrix component of blood vessels, *Col4a1* was expressed higher in tif-HEC than in T1 pre-HSC, further confirming the vascular endothelial property of the HECs we identified^32^ (Fig. 3f). In comparison, the genes enriched in PC2-negative direction mainly related to RNA splicing and blood coagulation (Fig. 3e). Together with the overrepresented *Spi1* in T1 pre-HSC (Fig. 3f), the data suggested that hematopoietic activity has been activated in T1 pre-HSC as compared to HEC.

The developmental path from tif-HEC to T1 pre-HSC was inferred by Mpath trajectory analysis (Fig. 3g). Consistently, during the course of in vitro culture of the PK44 population isolated from E10.0 AGM region on OP9-DL1 stromal cells to induce its HSC activity, we could witness the generation of immunophenotypic T1 pre-HSCs (Fig. 3h). The finding was in line with the sequential peaking of HECs and pre-HSCs, with the number of the latter reported to gradually increase from E10 and reach the peak at E11.^12,33^ We also evaluated the endothelial and hematopoietic potentials of the T1 pre-HSCs (CD31^+^CD45^−^CD41^low^Kit^+^CD201^high^) in E11.0 AGM region at single-cell level. Surprisingly, we found that although displayed largely decreased endothelial potential as compared to E10.0 PK44 cells (Fig. 2j), T1 pre-HSCs still maintained comparable endothelial-hematopoietic dual potential as that in PK44 population (Fig. 3i). This finding implied that the extremely rare and functionally enriched T1 pre-HSC population has not completely fulfilled the endothelial-to-hematopoietic fate transition.

### Enrichment of the HSC-competent HECs by newly established Neurl3-EGFP reporter

**Fig. 4:**
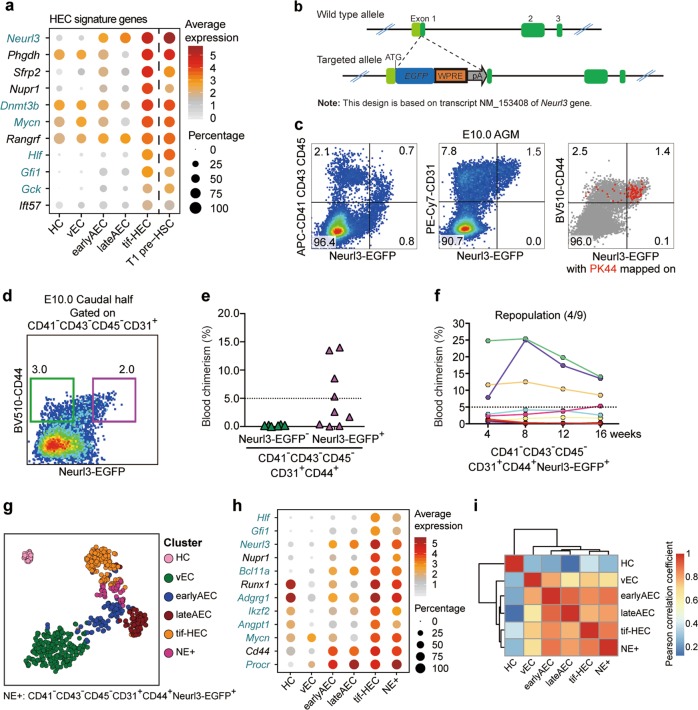
Identifying Neurl3 as a signature gene of HSC-primed HECs validated by functional and transcriptomic evaluation. **a** Dot plot showing the average and percentage expression of HEC signature genes in the indicated clusters. Genes are ordered by their median expression level in tif-HEC. Pre-HSC signature genes are marked as aquamarine. **b** Schematic model of the gene-targeting strategy for generating *Neurl3*^*EGFP/+*^ reporter mouse line via CRISPR/Cas9 system. **c** Representative FACS analysis of the E10.0 AGM region in *Neurl3*^*EGFP/+*^ embryos. FACS plot on the right shows PK44 cells (red dots) mapped on it. **d** Representative FACS plot for sorting of the indicated cell populations from E10.0 caudal half of *Neurl3*^*EGFP/+*^ embryos for the subsequent co-culture and transplantation assay. **e** Graph showing the donor chimerism at 16 weeks after transplantation of the derivatives of the indicated populations from the caudal half of E10.0 *Neurl3*^*EGFP/+*^ embryos. **f** Graph showing the donor chimerism at 4–16 weeks post-transplantation. The recipients were transplanted with the derivatives of CD41^−^CD43^−^CD45^−^CD31^+^CD44^+^Neurl3-EGFP^+^ population from the caudal half of E10.0 *Neurl3*^*EGFP/+*^ embryos. Number of repopulated/total recipients is shown in the brackets. **g** t-SNE plot of the cells included in the filtered initial dataset and additional PK44 and NE+ datasets, with clusters mapped on it. The HEC and PK44 clusters are combined as tif-HEC. NE+, CD41^−^CD43^−^CD45^−^CD31^+^CD44^+^Neurl3-EGFP^+^ population from E10.0 AGM region. **h** Dot plot showing the average and percentage expression of selected HEC feature genes in the indicated clusters. Pre-HSC signature genes are marked as aquamarine. **i** Heatmap showing the correlation coefficient between each two clusters with hierarchical clustering using average method. Pearson correlation coefficient is calculated using average expression of highly variable genes in each cluster.

In an effort to search for single marker to distinguish HSC-primed HECs from non-HECs or those CD45^−^ hematopoietic cells sharing an endothelial immunophenotype, we computationally screened for genes significantly overrepresented in the HEC cluster as compared to each of the other four clusters, including one hematopoietic cluster (HC) and three vascular EC clusters (vEC, earlyAEC and lateAEC) (Supplementary information, Fig. S1f). Totally eleven genes were screened out, which were then designated as signature genes of HSC-primed HECs, including three TFs (*Mycn*, *Hlf* and *Gfi1*) but no cell surface markers (Fig. 4a; Supplementary information, Table S5). Most of them manifested similarly high expression in T1 pre-HSCs, with six of them, namely *Neurl3*, *Dnmt3b*, *Mycn*, *Hlf*, *Gfi1*, and *Gck*, belonging to pre-HSC signature genes^11^ (Fig. 4a).

To further validate the bioinformatics findings and precisely determine the localization of these HSC-primed HECs, we specially chose *Neurl3* to establish a fluorescence reporter mouse line. As the median expression of *Neurl3* was the highest among these HEC signature genes, it was more likely to ensure enough sensitivity (Fig. 4a; Supplementary information, Fig. S3a and Table S5). By CRISPR/Cas9-mediated gene knockin strategy, the *EGFP* was inserted into the translational initiation codon of mouse *Neurl3* gene to ensure that EGFP would be expressed in exactly the same way as Neurl3 (Fig. 4b). We first evaluated the Neurl3-EGFP expression by flow cytometric analysis. At E10.0 AGM region, about half of the Neurl3-EGFP^+^ cells were hematopoietic (CD41/CD43/CD45-positive) cells, which constituted about one fourth of the hematopoietic population (Fig. 4c). All the Neurl3-EGFP^+^ cells were CD31^+^, and nearly all of them expressed CD44, indicative of the predominant aortic localization of Neurl3-EGFP^+^ ECs (Figs. 2b, 4c). Importantly, most of PK44 cells were Neurl3-EGFP^+^, highly suggesting the enrichment of HSC-competence by the Neurl3-EGFP^+^ ECs (Fig. 4c). To confirm the HSC-competence of the Neurl3-EGFP-labeled ECs, we performed co-culture plus transplantation assay using E10.0 Neurl3-EGFP mouse embryos (Fig. 4d). Although both could generate hematopoietic clusters under the in vitro culture condition, all the long-term (16 weeks) repopulations were detected exclusively in the recipients transplanted with the derivatives from CD44^+^Neurl3-EGFP^+^ ECs but not from CD44^+^Neurl3-EGFP^−^ ECs (Fig. 4e, f; Supplementary information, Fig. S3b).

We next investigated the transcriptomic identity of the Neurl3-EGFP^+^ ECs. We isolated ECs with an immunophenotype of CD41^−^CD43^−^CD45^−^CD31^+^CD44^+^Neurl3-EGFP^+^ (NE+) from E10.0 AGM region and performed scRNA-seq with totally 48 of them. All the sequenced NE+ cells that passed quality control ubiquitously expressed EGFP as expected and most of them expressed *Nerul3* and *Runx1* (Supplementary information, Fig. S3c–e). They distributed close to tif-HEC and were predominantly located between tif-HEC and earlyAEC by t-SNE visualization (Fig. 4g; Supplementary information, Table S1). Accordingly, NE+ cells demonstrated the increased cycling as compared to earlyAEC, presenting an intermediate proliferative status between earlyAEC and tif-HEC (Supplementary information, Fig. S3f). Similar to tif-HEC, NE+ cells showed relatively high expression of a set of HEC feature genes and pre-HSC signature genes (Figs. 2i, 4h). Correlation analysis revealed that NE+ cells showed the highest similarity with tif-HEC and they were clustered together by hierarchical clustering, whereas earlyAEC and lateAEC were much correlated (Fig. 4i). Therefore, from the functional and transcriptomic evaluation, the performance of the Neurl3-EGFP-marked ECs was basically consistent with the prediction of HSC-primed HEC by unsupervised computational screening.

### In situ localization and in vitro function of the HECs marked by Neurl3-EGFP reporter

**Fig. 5:**
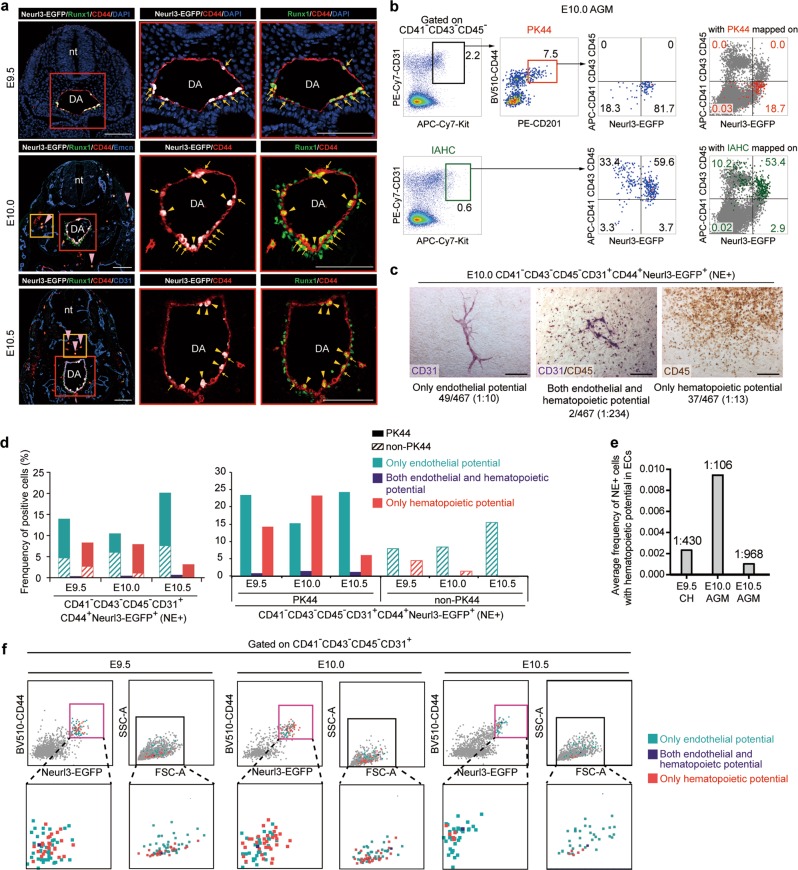
In situ localization and in vitro function of the dynamic HECs marked by Neurl3-EGFP reporter. **a** Representative immunostaining on cross sections at the AGM region of E9.5 (upper), E10.0 (middle) and E10.5 (lower) *Neurl3*^*EGFP/+*^ embryos. Arrows indicate Neurl3-EGFP^+^ aortic ECs. Yellow arrowheads indicate Neurl3-EGFP^+^ bulging and bulged cells and also IAHCs. White pink arrowheads indicate CD44^+^Runx1^+^Neurl3^−^ hematopoietic cells distributed outside the aorta. The high magnification views of yellow boxes are shown in Supplemental information, Fig. S3g. nt, neural tube; DA, dorsal aorta. Scale bars, 100 μm. **b** Representative FACS analysis of the E10.0 AGM region of *Neurl3*^*EGFP/+*^ embryos. FACS plots on the right showing PK44 cells (red dots, upper) and IAHC (CD31^+^Kit^high^) cells (green dots, lower) mapped on, respectively, with their contributions to the population in each gated quadrant indicated. **c** Representative CD31 and CD45 immunostaining on the cultures of single NE+ (CD41^−^CD43^−^CD45^−^CD31^+^CD44^+^Neurl3-EGFP^+^) cells from E10.0 AGM region of *Neurl3*^*EGFP/+*^ embryos, showing typical morphologies regarding distinct differentiation potentials. Cell frequencies of each kind of potential are also shown. Data are from 5 independent experiments with totally 37 embryos used. Scale bars, 400 μm. **d** Column charts showing the frequencies of positive cells in the indicated populations (lower) for each kind of potential. The experiments were performed with NE+ (CD41^−^CD43^−^CD45^−^CD31^+^CD44^+^Neurl3-EGFP^+^) single cells from E9.5 caudal half or E10.0-E10.5 AGM region of *Neurl3*^*EGFP/+*^ embryos with PK44 indexed. Progenies from PK44 and non-PK44 fractions within NE+ cells are represented by distinct fill patterns. **e** Graph showing the average frequencies of the NE+ (CD41^−^CD43^−^CD45^−^CD31^+^CD44^+^Neurl3-EGFP^+^) cells with hematopoietic potential in ECs (CD41^−^CD43^−^CD45^−^CD31^+^) in E9.5 caudal half (CH) or E10.0–E10.5 AGM region of *Neurl3*^*EGFP/+*^ embryos. **f** Expression of CD44 and Neurl3-EGFP and values of FSC-A and SSC-A in the index-sorted single NE+ (CD41^−^CD43^−^CD45^−^CD31^+^CD44^+^Neurl3-EGFP^+^) cells with differentiation potential based on in vitro functional evaluation. Cells with different kinds of potentials are mapped onto the reference FACS plots (gray dots). Pink boxes indicate the gates of the populations for FACS sorting. The enlarged views of solid boxes are shown below.

At the AGM region of E9.5–E11.0 embryos, CD44 expression marked the whole endothelial layer of dorsal aorta in addition to IAHC cells, in line with the whole mount staining (Figs. 2b, 5a; Supplementary information, Fig. S3g). Of note, Neurl3-EGFP expression was restricted to the IAHCs and partial aortic ECs, where Neurl3-EGFP and Runx1 presented a highly co-expression pattern (Fig. 5a; Supplementary information, Fig. S3g). Thus the Neurl3-EGFP^+^ cells embedded in the endothelial layer largely enriched the putative HECs. By FACS analysis, the average constitution of Neurl3-EGFP^+^ cells in CD44^+^ ECs were 35.7%, 50.2% and 17.7% in E9.5 caudal half, E10.0 and E10.5 AGM region, respectively (Supplementary information, Fig. S3h). Considering the slight over-estimation due to the high sensitivity of FACS, the data were basically in accordance with the morphological findings (Fig. 5a) and the estimated HEC composition by scRNA-seq (Supplementary information, Fig. S1f). The temporal dynamics of the HEC we defined here was also in line with that of Runx1 expression in aortic endothelial layer reported.^34^ The HEC population peaked at E10.0, about 0.5 days earlier than the time point when the number of IAHC cells reaches the peak and the first HSCs are detected in AGM region.^7,27^ Of particular note, the expression of Neurl3-EGFP was completely absent from the sub-aortic mesenchyme, in contrast to the widespread distribution of Runx1 there at E10.0-E11.0 (Fig. 5a; Supplementary information, Fig. S3g). Although scattered Runx1^+^CD44^+^ round blood cells were easily witnessed, much fewer Neurl3-EGFP-expressed cells outside dorsal aorta were detected, even at E11.0 (Fig. 5a; Supplementary information, Fig. S3g).

Given the lack of suitable antibodies to directly determine the anatomical localization of PK44 cells, which have been proven as HSC-competent HECs (Fig. 2c–e), we compared in FACS plots the distributions of PK44 and Neurl3-EGFP^+^ cells, whose in situ localization was clearly defined (Fig. 5a), with the CD31^+^Kit^high^ population (known as IAHC cells) as a parallel reference.^7^ In E10.0 AGM region, about 80% of PK44 cells and 60% of IAHC cells were Neurl3-EGFP^+^, with most CD31^+^Kit^high^ cells being CD41/CD43/CD45-positive hematopoietic cells as previously reported^7^ (Fig. 5b; Supplementary information, Fig. S3i). PK44 showed an expression pattern largely different from CD31^+^Kit^high^ cells, suggestive of its predominant endothelial layer rather than IAHC localization (Fig. 5b).

As less than half of Neurl3-EGFP^+^ ECs showed a PK44 immunophenotype in E10.0 AGM (Fig. 5b; Supplementary information, Fig. S3i), we next explored the in vitro functional relationship of PK44 and non-PK44 fractions within NE+ cells (CD41^−^CD43^−^CD45^−^CD31^+^CD44^+^Neurl3-EGFP^+^) by index-sorting. From E9.5 to E10.5, all three kinds of potentials, including endothelial-only, hematopoietic-only, and endothelial-hematopoietic dual potential, could be detected in NE+ cells, with different frequencies (Fig. 5c, d; Supplementary information, Fig. S3j). The average frequency of the NE+ cells with hematopoietic potential in ECs peaked at E10.0 and apparently decreased at E10.5 (Fig. 5e). The potential was remarkably biased to endothelial at E10.5 as compared to E9.5 and E10.0 (Fig. 5d), which should be due to the prompt loss of Neurl3-EGFP-labeled HECs and the possible labeling of some lateAECs by Neurl3-EGFP (Fig. 4a, h). Of note, all three kinds of potentials were obviously higher in PK44 than non-PK44 fraction, with endothelial-hematopoietic dual potential exclusively detected in PK44 cells (Fig. 5d). Therefore, presenting a PK44 immunophenotype represented the enriched functional sub-populations within Neurl3-EGFP^+^ HECs (Fig. 5d; Supplementary information, Fig. S3i). All three kinds of potentials did not show an evidently biased distribution regarding CD44 or Neurl3-EGFP expression level by index sorting analysis (Fig. 5f). Interestingly, cells with the hematopoietic rather than endothelial potential intended to have smaller side scatter density on FACS (Fig. 5f).

### Stepwise fate choices of HSC-primed HECs from primitive vascular ECs

In an effort to decipher the stepwise specification of the HSC-primed HECs, we added the immunophenotypic EC samples, from the stage of initial aortic structure formation at E8.0^35^ to E9.0, to achieve seamless sampling at continuous developmental stages (Supplementary information, Fig. S4a). All the transcriptomically identified ECs were re-clustered into six clusters, with four of them basically consistent with those previously defined, namely vEC, earlyAEC, lateAEC, and HEC. The newly added samples were mainly distributed into three clusters, vEC, primitive EC (pEC) featured by *Etv2* expression and involving almost all E8.0 cells, and primitive arterial EC (pAEC) given the expression of arterial marker *Gja5* and serving as the earliest arterial EC population (Supplementary information, Fig. S4b, c). The latter two clusters were newly identified.

**Fig. 6:**
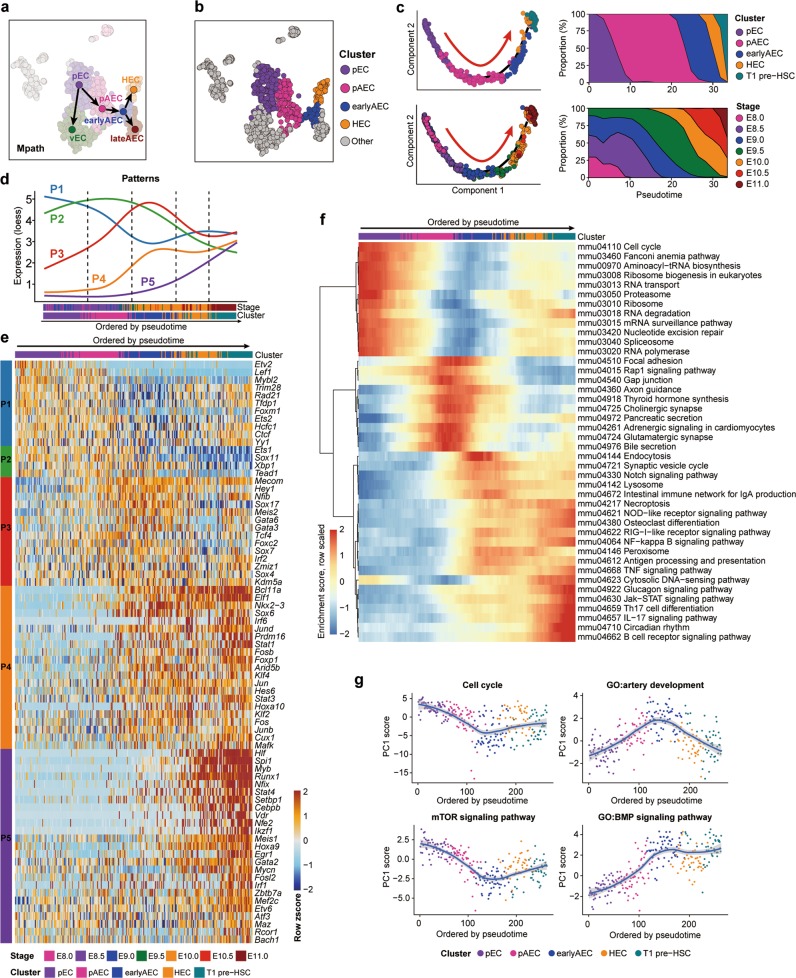
Molecular evolution underlying the specification of HSC-primed HECs from primitive vascular ECs. **a** Trajectories of pEC, vEC, pAEC, earlyAEC, lateAEC and HEC inferred by Mpath. Arrows indicate the development directions predicted by sampling stages. **b** t-SNE plot showing the distribution of the four clusters involved in hemogenic specification. Other cells are in gray. **c** Pseudotemporal ordering of the cells included in the indicated five clusters inferred by monocle 2 (left), with clusters (upper left) and sampling stages (lower left) mapped to it. HEC specification directions are indicated as red arrows. Smooth distributions of clusters (upper right) and sampling stages (lower right) along pseudotime by using Gaussian kernel density estimate are shown. **d** Dynamic changes of five gene expression patterns along the trajectory ordered by pseudotime inferred by monocle 2. For each pattern, principal curves are fitted on expression levels of the genes in that pattern along pseudotemporal order, using local polynomial regression fitting method. Randomly down-sampling is performed in pEC and pAEC clusters for better visualization. **e** Heatmap showing the relative expression of the core TFs belonging to the regulons, genes in which exhibit significant overlap with the pattern genes. Cells are ordered by pseudotime and TFs are ordered by Patterns. **f** Heatmap showing smoothed (along adjacent 25 cells) and scaled enrichment scores of top 50 KEGG pathways along the order by pseudotime. Pathways are ordered by hierarchical clustering using ward.D method. **g** Scatter plots showing the relative activity levels of pathways or GO terms with loess smoothed fit curves and 95% confidence interval indicated. Relative activity levels are represented by the PC1 scores of expression levels of the genes in a given set. The sign or direction of PC1 is corrected according to positive correlation with averaged expression levels.

Trajectory analysis by Mpath demonstrated two bifurcations along the path from pEC to HEC and revealed a predominant two-step fate choice (Fig. 6a). pEC first chose an arterial but not venous fate to become pAEC, then upon maturing into earlyAEC and lateAEC, HEC chose to segregate from the intermediate arterial population earlyAEC (Fig. 6a), in line with the finding that the HEC displayed certain arterial characteristics but was completely devoid of venous feature (Fig. 1h). To decipher the underlying molecular programs for HEC specification, we specifically selected four clusters, excluding vEC and lateAEC branched out from the path from pEC to HEC, and added T1 pre-HSC as the end point for the subsequent analysis (Fig. 6b). Monocle 2 elegantly recapitulated the sequential sampling stages and the deduced cellular evolution upon stepwise hemogenic specification along the inferred pseudotime (Fig. 6c; Supplementary information, Fig. S4d).

We identified totally 2851 genes whose expression levels were changed significantly among five clusters, which were further grouped into five principal expression patterns along the inferred pseudotime (Fig. 6d; Supplementary information, Fig. S4e and Table S6). In general, the expressions of the genes in Pattern 1 showed the highest in pEC, decreased apparently upon arterial specification, whereas slightly increased upon hemogenic specification. These genes were mainly related to rRNA processing and mitotic nuclear division (Fig. 6d; Supplementary information, Fig. S4e, f). Genes in Pattern 2 and Pattern 3 showed the highest expression in pAEC and earlyAEC, respectively, and were subsequently down-regulated (Fig. 6d). They were related to actin filament organization and cell migration, respectively, indicative of the employment of different behaviors at different maturation status of arterial ECs (Supplementary information, Fig. S4e, f). Genes in Pattern 4 and Pattern 5 both had the highest expression in the final T1 pre-HSC and both exhibited hematopoiesis-related terms. Expression of genes in Pattern 4 reached the relatively high level at the earlyAEC stage, whereas that of Pattern 5 genes showed a gradual increase (Fig. 6d; Supplementary information, Fig. S4e, f).

Among the above 2851 pattern genes, 75 TFs belonged to the core TFs of the regulons where the genes included significantly overlapped with the pattern genes (Fig. 6e; Supplementary information, Table S6). Given the simultaneous co-expression of the core TF and its predicted targets in a given regulon, these core TFs were considered to presumably play a role to drive or orchestrate the dynamic molecular program during HEC specification (Fig. 6e). Most of these TFs belonged to Pattern 4 and Pattern 5, indicating that most activated TFs along HEC specification from primitive vascular ECs were those overrepresented in the final hemogenic and hematopoietic populations (Fig. 6e). We also examined the expression patterns of totally 28 TFs previously reported to have a role in HSPC regeneration in vitro.^36–39^ Nineteen of these presumed functional TFs were dynamically changed, among which 15 were core TFs of regulons and 13 belonged to Pattern 5 (Supplementary information, Fig. S4g).

We next evaluated the pathway enrichment for each cell to depict dynamic changes at pathway level. The enrichment of pathways differently activated among the five candidate clusters showed the dynamic patterns similar to gene expression patterns (Fig. 6f). Among these pathways, those associated with cell cycle, ribosome and spliceosome were down-regulated with arterial specification whereas turned to be moderately up-regulated upon hemogenic specification (Fig. 6f, g). In contrast, several pathways experienced a completely opposite change, such as the Rap1 signaling pathway (Fig. 6f). The activities of pathways related to artery development, and its pivotal executor the Notch signaling pathway,^2,40^ first rose to peak in earlyAEC and then modestly fell down upon hemogenic specification (Fig. 6f, g). Some inflammation-related pathways, including the NFκB and TNF signaling pathways, were activated from earlyAEC to the final T1 pre-HSC, in line with the notion about the requirement of inflammatory signaling during HSC generation^41^ (Fig. 6f).

## Disccussion

Here via unbiasedly going through all the relevant EC populations, HSC-primed HECs were transcriptomically identified. More importantly, combining the computational prediction and in vivo functional evaluation, we precisely captured the HSC-competent HECs by a newly constructed fluorescent reporter mouse model, Neurl3-EGFP, and revealed further functionally enriched sub-population within Neurl3-EGFP-labeled ECs by a set of surface marker combination PK44 (Fig. 5d; Supplementary information, Fig. S3i). Together with our most recent report showing CD201 marks all the HSCs in AGM region by direct transplantation,^42^ our findings thus pinpoint CD201 as a powerful enriching surface marker for the whole process of HEC-to-HSC transition.^11^ Serving as the putative marker of HSC-primed HECs,^14,22^ *Gfi1* was specifically expressed in HEC but not other EC-related populations (Fig. 4h), supportive of the cluster assignment. Belonging to the gene family of E3 ubiquitin ligases, the expression and role of Neurl3 in spermatogenesis and inflammation have been reported,^43–45^ whereas that relevant to vascular and hematopoietic development remain barely known. Neurl3 was screened out by unsupervised bioinformatics analysis, and fortunately, its expression in the AGM region was restricted to aorta and largely consistent with that of *Runx1* both transcriptomically (Supplementary information, Fig. S1k) and anatomically (Fig. 5a) regarding endothelial expression. Although highly expressed in tif-HEC, *Runx1* and *Adgrg1* were also highly expressed in the CD45^−^ hematopoietic population (Fig. 4h), which should be the derivatives of HSC-independent hematopoiesis. This suggested that they may not distinguish the precursors of HSCs and non-HSCs,^34,46^ thus *Runx1* and *Adgrg1* were not included in the list of the signature genes of HSC-primed HEC (Fig. 4a). The specificity of *Nerul3* expression related to HSC generation suggests that the Neurl3-EGFP would be a good reporter for the studies of HSC development.

Based on the in vivo functional validation of the HSC-primed HECs and the sampling of continuous developmental stages with intervals of 0.5 days, we had a good opportunity to evaluate the dynamics and functional heterogeneity of these important transient populations. Unexpectedly, the HSC-competent HECs we identified here demonstrated a previously unresolved endothelial-hematopoietic dual potential. Marked by either PK44 or Neurl3-EGFP, the HECs showed higher enrichment of hemogenic properties both transcriptomically and functionally than those using Runx1 +23GFP as a maker,^3^ including the expression of key hematopoietic TFs (Supplementary information, Figs. S2d and S3d) and the in vitro hematopoietic or endothelial potential (Supplementary information, Figs. S2f and S3j), which might partially explain why the rare endothelial-hematopoietic dual potential is hardly detected around the timing of HSC emergence in the previous report.^3^ Thus, our findings well supplement the functional evaluation of putative HECs, which have a dynamic and transient nature; without catching the endothelial-hematopoietic dual potential, it is hard to define a given population being experiencing the endothelial-to-hemogenic fate conversion. Both the constitution and the hemogenic potential of the HSC-primed HECs reached the peak at the time point about 0.5 days before the first HSC emergence, and rapidly decreased thereafter (Fig. 5e; Supplementary information, Figs. S1f and S3h). Interestingly, the endothelial-hematopoietic dual potential was still maintained until T1 pre-HSC stage at E11.0 (Fig. 3i), when cells have begun to express hematopoietic surface markers (Fig. 3b) and turned to be round in shape.^15^ The data suggest that the hematopoietic fate might not have been fixed in T1 pre-HSC, which needs further investigations.

We also precisely decoded the developmental path of HSC-primed HECs from the initially specified vascular ECs, the view of which has been generally neglected previously. We found that the gene expressions and the activities of pathways involved in arterial development and the Notch signaling first increased and then decreased upon HEC specification (Fig. 6e–g). Supportively, several seemingly contradictory findings have been reported regarding the role of the Notch signaling in HEC specification. For example, activation of arterial program or the Notch signaling is known to be required for HEC specification in mouse embryos or generation of HECs with lymphoid potential from human pluripotent stem cells.^47–49^ On the other hand, repression of arterial genes in EC after arterial fate acquisition leads to augmented hematopoietic output.^50^ Noteworthy, we revealed two bifurcates during HSC-primed HEC specification along the path from primitive vascular EC, suggesting two-step fate choice occurred for hemogenic fate settling (Fig. 6a). Serving as the two presumed final fates of earlyAEC, HEC and lateAEC displayed a series of differences (Fig. 1l), which better explains the presumably misinterpreted notion in previous report that arterial ECs and HSCs originate from distinct precursors.^18^ Our findings further emphasize that arterial specification and the Notch signaling activation should be precisely and stepwise controlled for HSC generation. Although both showing obvious similarities regarding the arterial features and anatomical distributions, the molecular difference between earlyAEC and lateAEC should also be paid attention to as the former but not the latter is likely to be the direct origin of the HSC-primed HECs.

We also revealed several similarities regarding the molecular events underlying the development of HSC-primed HECs between mouse embryos in the present study and human embryos that we have reported very recently,^19^ including the arterial features and the overrepresented ribosome and translational activities in the HSC-primed HECs. Such conservation further assures the mouse model as an ideal animal model for HSC development studies. Interestingly, we have identified two transcriptomically different types of intra-embryonic HECs in human embryos at different developmental stages, and the earlier one is mainly detected at Carnegie stage 10 (equal to E8.5–E9.0 in mouse embryos).^19^ However, this earlier wave of intra-embryonic HECs was not observed in mouse embryos in the present study (Fig. S4b), suggesting that the contribution of distinct hemogenic sites to the emergence of HSC-independent hematopoiesis might be different in mouse and human embryos. Taking advantage of mouse models and by using either surface marker combination PK44 or the newly established Neurl3-EGFP reporter to enrich the HEC population, we revealed here the unambiguous in vivo repopulating potential of HSC-primed HECs after in vitro co-culture and the extremely rare endothelial-hematopoietic dual potential of single cells within the enriched HEC population, which have not been achieved in human samples.

The comprehensive understanding of cellular evolutions and molecular programs underlying the specification of HSC-primed HECs combined with the important spatiotemporal cues in vivo will facilitate future investigations directing HSC formation in vitro and other related regeneration strategies. For example, multiple previously unknown TFs that might potentially play a role in the hemogenic specification from ECs (Fig. 6e) could be tried individually or in combination in the strategies of in vitro HSPC induction from pluripotent stem cells. In addition, our findings further suggest the employment of multi-step induction after the initial EC generation in such culture systems, in which hemogenic induction following but not simultaneous with the arterial induction is worth trying. Moreover, as a single marker capable of enriching the HSC-primed HECs, the novel Neurl3-EGFP reporter would further facilitate the high-throughput screening in the studies on HSPC induction from pluripotent stem cells.

## Materials and methods

### Mice

Mice were handled at the Laboratory Animal Center of Academy of Military Medical Sciences in accordance with institutional guidelines. Mouse manipulations were approved by the Animal Care and Use Committee of the Institute. The *Neurl3*^*EGFP/+*^ reporter mouse lines were generated with the CRISPR/Cas9 technique by Beijing Biocytogen. All mice were maintained on C57BL/6 background. Embryos were staged by somite pair (sp) counting: E8.0, 1–7 sp; E8.5, 8–12 sp; E9.0, 13–20 sp; E9.5, 21–30 sp; E10.0, 31–35 sp; E10.5, 36–40 sp; and E11.0, 41–45 sp. In some experiments, caudal half of E10.0 embryo was dissected under heart with limbs removed. AGM region was dissected as reported.^12^ The fluorescent dye Oregon green 488 was purchased from Invitrogen. Staining was performed as previously described^12^ except that the concentration of staining solution was 5 μmol/L and the time of staining was 3 min before washed. Primary embryonic single-cell suspension was acquired by type I collagenase digestion.

### Flow cytometry

Cells were sorted and analyzed by flow cytometers FACS Aria 2 and Calibur (BD Biosciences), and the data were analyzed using FlowJo software (Tree Star). Cells were stained by the following antibodies: B220 (eBioscience, RA3-6B2), CD3 (eBioscience, 145-2C11), CD4 (eBioscience, GK1.5), CD8a (eBioscience, 53-6.7), CD31 (BD or BioLegend, MEC13.3), CD41 (BD or eBioscience, MWReg30), CD43 (BD, S7), CD44 (eBioscience or BioLegend, IM7), CD45.1 (eBioscience, A20), CD45.2 (eBioscience, 104), CD45 (eBioscience, 30-F11), CD144 (eBioscience, eBioBV13), CD201 (eBioscience, eBio1560), Flk1 (eBioscience, Avas12a1), Kit (eBioscience, 2B8), Ly-6G (BioLegend, 1A8), and Mac-1 (eBioscience, M1/70). 7-amino-actinomycin D (7-AAD; eBioscience) was used to exclude dead cells. For index sorting, the FACS Diva 8 “index sorting” function was activated and sorting was performed in single-cell mode.

### Colony forming unit-culture (CFU-C) assay

Cells were sorted by flow cytometry and plated in 35 mm Petri dish containing 2 mL methylcellulose-based medium with recombinant cytokines (MethoCult GF M3434, STEMCELL Technologies). The cells were incubated for 7 days for colony quantification.

### OP9-based hematopoietic and endothelial potential assay

Cells were sorted by flow cytometry in single-cell mode and were then plated on the OP9 or OP9-DL1 stromal cells^51^ in IMDM (Hyclone) containing 15% fetal bovine serum (Hyclone), 1% bovine serum albumin (Sigma), 10 μg/mL insulin (Macgene), 200 μg/mL transferrin (Sigma), and 5.5 × 10^−5^ mol/L 2-mercaptoethanol (Gibco). For the endothelial potential assay, 100 ng/mL rhVEGF-165 (PeproTech) was supplemented. For hematopoietic and endothelial dual potential assay with 10 cells or single cell plated per well, both 100 ng/mL rhVEGF-165 and 50 ng/mL SCF (PeproTech) were supplemented. After 7 days of co-culture, cells were fixed in 4% paraformaldehyde for 30 min and stained with PE-conjugated or purified CD45 antibody (eBioscience, 30-F11 or BD Biosciences) to ascertain the generation of hematopoietic progeny. Subsequently, CD31 (BD Pharmingen, MEC13.3) immunohistochemistry staining was performed using standard procedures, and the formation of CD31-positive tubules in the wells was considered as having endothelial potential.

### OP9-DL1 co-culture and transplantation assay

To investigate the HSC potential of the presumed HSC-primed HEC populations in E9.5–E10.0 caudal half, CD45.1/2 embryos were used as donor for PK44 cells and *Neurl3*^*EGFP/+*^ embryos (CD45.2/2 background) for NE+ cells. FACS-purified cell populations were plated on the OP9-DL1 stromal cells in α-MEM (Gibco) supplemented with 10% fetal bovine serum (Hyclone) and cytokines (100 ng/mL SCF, 100 ng/mL IL-3 and 100 ng/mL Flt3 ligand, all from PeproTech). After 7 days of co-culture, cells were harvested and then injected into 8–12 weeks female recipients (CD45.2/2 background for PK44 cells and CD45.1/2 for NE+ cells) via tail vein, along with 2 × 10^4^ nucleated fresh bone marrow carrier cells (CD45.2/2 background for PK44 cells and CD45.1/1 for NE+ cells) per recipient. Recipients were pre-treated by a split dose of 9 Gy γ-irradiation (^60^Co). Peripheral blood cells of recipients were analyzed by flow cytometry at the indicated time points to determine the chimerism. The recipients demonstrating ≥ 5% donor-derived chimerism in CD45^+^ cells of peripheral blood were considered as successfully reconstituted. Multi-organ and multi-lineage reconstitution were evaluated as reported.^52^ Totally 1 × 10^7^ bone marrow cells obtained from the reconstituted primary recipients at 16 weeks post-transplantation were injected into the secondary recipients to investigate HSC self-renewal potential.

### Immunofluorescence

Embryos were isolated, fixed with 4% paraformaldehyde for 30 min to 2 h at 4 °C, embedded in paraffin, and sectioned at 5–6 μm with Leica RM2235. Sections were deparaffinized with ethanol of gradient concentration, then blocked in blocking solution (Zhongshan golden bridge) for 30 min at room temperature, followed by incubation with primary antibodies overnight at 4 °C. After 3 washes (3 min each) in PBS, sections were incubated with corresponding secondary antibodies (Zhongshan golden bridge) for 30 min at room temperature. After 3 washes in PBS, sections were stained with DendronFluor TSA (Histova, NEON 4-color IHC Kit for FFPE, NEFP450, 1:100, 20–60 s). The primary and secondary antibodies were thoroughly eluted by heating the slides in citrate buffer (pH 6.0) for 20 min at 95 °C using microwave. In a serial fashion, each antigen was labeled by distinct fluorophores. After all the antibodies were detected sequentially, the slices were finally stained with DAPI. Images were collected by confocal microscope (Nikon Ti-E A1/ ZEISS LSM 880). The primary antibodies were as follows: CD31 (BD Biosciences), CD44 (BD Biosciences), Endomucin (eBioscience), GFP (Cell Signaling), and Runx1 (Abcam).

### Whole-mount immunofluorescence

The body part between forelimb buds and hindlimb buds of E10.0 embryo was dissected, fixed in 2% PFA/PBS for 20 min on ice and dehydrated in graded concentrations of methanol/PBS (50%, 100%; 10 min each). To block endogenous peroxidase, samples were bleached in 5% H_2_O_2_ for 1 h on ice. For staining, the samples were blocked in PBSMT (1% skim milk and 0.4% Triton X-100 in PBS) containing 0.2% BSA for 1 h at 4 °C, incubated with PBSMT containing anti-CD44 (1:25) overnight at 4 °C, then washed 3 times in PBSMT each for 1 h at 4 °C. The primary antibody was developed by incubating HRP-conjugated anti-rat IgG antibody (1:2000 in PBSMT; Zhongshan golden bridge) overnight at 4 °C. After extensive washing with more than 3 exchanges of PBSMT, including the final 20 min wash in PBST (0.4% Triton X-100 in PBS) at 4 °C, the samples were soaked in DendronFluor TSA (Histova, NEON 4-color IHC Kit for Wholemount/Cytometry, NEWM450) for 10–30 min, and hydrogen peroxide was added to 0.03%. The enzymatic reaction was allowed to proceed until the desired color intensity was reached, and the samples were rinsed 3 times in PBST. Finally, the samples were dehydrated in 100% methanol, soaked in graded concentrations of BABB (phenylcarbinol and benzyl benzoate, 1:2)/methanol (50%, 100%; 1 min each), and stored at −20 °C until photographed.

### Single-cell RNA-seq library construction

Single cells in good condition were picked into lysis buffer by mouth pipetting. The scRNA-seq preparation procedure was based on STRT with some modifications.^53–55^ cDNAs were synthesized using sample-specific 25 nt oligo dT primer containing 8 nt barcode (TCAGACGTGTGCTCTTCCGATCT-XXXXXXXX-NNNNNNNN-T25, X representing sample-specific barcode whereas N standing for unique molecular identifiers, UMI, see Supplementary information, Table S7) and TSO primer for template switching.^56–58^ After reverse transcription and second-strand cDNA synthesis, the cDNAs were amplified by 17 cycles of PCR using ISPCR primer and 3’ Anchor primer (see Supplementary information, Table S7). Up to 56 samples were pooled and purified using Agencourt AMPure XP beads (Beckman). Four cycles of PCR were performed to introduce index sequence (see Supplementary information, Table S7). After this step, 400 ng cDNAs were fragmented to around 300 bp by covaris S2. The cDNA was incubated with Dynabeads MyOne^TM^ Streptavidin C1 beads (Thermo Fisher) for 1 h at room temperature. Libraries were generated using KAPA Hyper Prep Kit (Kapa Biosystems). After adapter ligation, the libraries were amplified by 7 cycles of PCR using QP2 primer and short universal primer (see Supplementary information, Table S7). The libraries were sequenced on Illumina HiSeq 4000 platform in 150 bp pair-ended manner (sequenced by Novogene).

### Quantification of gene expression for scRNA-seq data

We used unique molecular identifier (UMI)-based scRNA-seq method to measure the gene expression profiles within individual cells. Raw reads were first split by specific barcode attached in Read 2 for individual cells and UMI information was aligned to the corresponding Read 1. Read 1 was trimmed to remove the template switch oligo (TSO) sequence and polyA tail sequence. Subsequently, quality control was conducted to discard reads with adapter contaminants or low-quality bases (N > 10%). Next, the mm10 mouse transcriptome (UCSC) was used to align the clean reads using TopHat (version 2.0.12).^59^ Uniquely mapped reads were obtained using HTSeq package^60^ and grouped by the cell-specific barcodes. Transcripts of each gene were deduplicated based on the UMI information, while mitochondrial genes were not included for quantification. Finally, for each gene in each individual cell, the number of the distinct UMIs derived from that gene was regarded as its copy number of transcripts.

### Quality control and normalization of sequencing data

For the 662 sequenced single cells from E9.5–E11.0 embryos of totally 29 embryos, we only retained cells with more than 2000 genes and 100,000 transcripts detected. Then, 597 cells passed the filter standards. Gene expression levels in each cell were normalized by log_2_(TPM/10 + 1), where TPM (transcripts-per-million) was calculated as (the number of UMIs of each gene/all UMIs of a given cell) ×1,000,000. Since the UMI number of most of our samples was less than the order of 1,000,000 transcripts, the TPM values were divided by 10 to avoid counting each transcript for several times. On average we detected 7035 genes (range from 2266 to 10,843) and 636,418 transcripts (range from 103,793 to 2,959,573) expressed in each individual cell.

Additionally, we also sequenced 96 single cells with a PK44 immunophenotype (CD41^−^CD43^−^CD45^−^CD31^+^CD201^+^Kit^+^CD44^+^) from E10.0 AGM regions of totally 9 embryos, 47 T1 pre-HSCs (CD31^+^CD45^−^CD41^low^Kit^+^CD201^high^) from E11.0 AGM regions of totally 18 embryos, 48 single cells with an immunophenotype of CD41^−^CD43^−^CD45^−^CD31^+^CD44^+^Neurl3-EGFP^+^ from Neurl3-EGFP reporter mouse embryos and 579 single cells from E8.0–E9.0 body regions of totally 24 embryos. The same quality control criteria and normalization method described above were applied to these additional datasets. In total, 1432 single cells were sequenced and 1325 cells passed the filter standards and were used for downstream analyses (see Supplementary information, Table S1).

### Dimensional reduction and clustering

We used Seurat R package (version 2.3.4)^61^ for further analyzes and exploration of our single cell RNA sequencing data, such as identification of highly variable genes (HVGs) and differentially expressed genes (DEGs), dimension reduction using PCA or t-SNE, unsupervised clustering and so on. A standard analysis process is briefly described below. First, only genes expressed in at least 3 single cells were retained so as to exclude genes that were hardly expressed. Then, FindVariableGenes function was used to select HVGs on log2 (TPM/10 + 1) transformed expression values. Genes with average expression more than 1 and less than 8 and dispersion greater than 1 were identified as HVGs. To mitigate the effect of cell cycle, HVGs not included in the direct cell cycle GO term (GO:0007049) (Supplementary information, Table S7) were used as inputs for PCA dimension reduction. Elbow method was employed to select the top relevant PCs for subsequent t-SNE dimension reduction and graph-based clustering.^28^

For the initial dataset from E9.5–E11.0 body and DA locations, we select top 15 PCs for clustering using FindClusters with default settings, to obtain 6 major clusters. Negative control cells with a non-EC immunophenotype and cells grouped with these negative control cells were reclassified specifically into the Neg cluster. The remaining cells were assigned as vEC, earlyAEC, lateAEC, HEC and HC clusters based on the clustering results. Next, cells in Neg cluster and cells with *Ptprc* or *Spn* expression level greater than 1 were removed. Then, the filtered initial dataset was used for analyzes of subdatasets, including subdividing of HEC cluster, subdividing of earlyAEC cluster and in-depth analyses of earlyAEC, lateAEC and HEC clusters. The filtered initial dataset was also included in three combined datasets of combining PK44 cell population, PK44 and T1 pre-HSC cell populations, and PK44 and Neurl3-EGFP cell populations, respectively. Dimension reduction and clustering analyses for subdatasets and combined datasets abovementioned also followed the same procedure as described above. See Supplementary information, Table S1 for detailed cell information.

For combined dataset from earlier dataset (E8.0–E9.0 body location) and initial dataset (E9.5–E11.0 body and DA locations), we redid the dimension reduction and clustering analyses over again. Same as the processing of initial dataset, negative control cells with a non-EC immunophenotype and cells grouped with these negative control cells were reclassified manually into Neg cluster. The remaining cells were assigned as vEC, pEC, pAEC, earlyAEC, lateAEC, HEC and HC based on the clustering results. The new clustering results are highly consistent with the previous ones within the common cell populations. Next, cells in Neg cluster and cells with *Ptprc* or *Spn* expression level greater than 1 were removed. Cells in pEC, pAEC, lateAEC and HEC and cells from T1 pre-HSC dataset were retained for further analysis.

### Identification of DEGs

DEGs were identified using FindMarkers or FindAllMarkers functions with default Wilcoxon rank sum test and only genes detected in a minimum fraction of 0.25 cells in either of the two populations were considered. Genes with fold-change ≥ 2 and adjusted *P* value ≤ 0.05 were selected as DEGs.

### Arterial and venous feature score

Arteriovenous marker genes previously known or inferred from the artery development pattern genes, including 10 arterial genes (*Dll4*, *Igfbp3*, *Unc5b*, *Gja4*, *Hey1*, *Mecom*, *Efnb2*, *Epas1*, *Vegfc* and *Cxcr4*) and 3 venous genes (*Nr2f2*, *Nrp2*, and *Aplnr*),^35,62–64^ were selected to perform the arteriovenous feature scores. First, we scaled the log_2_(TPM/10 + 1) expression values of each marker gene to 0–10 scale among all the sample cells after quality control. Second, for each cell, we averaged the scaled values of arterial genes and venous genes, respectively. Third, the averaged values were rescaled to 0–10 scale across all the sample cells to finally achieve the arterial and venous scores. For each population, the arterial and venous scores of all of the cells within the population were average. The 50% confidence ellipses were also calculated to show the main distribution ranges. We chose score value = 5 as the threshold to infer the arterial or venous identity of vascular ECs, as the distribution of individual cells was in line with the notion showing essentially no arterial/venous double positive cells.

### Cell cycle analysis

For cell cycle analysis, cell cycle-related genes consisting of a previously defined core set of 43 G1/S genes and 54 G2/M genes were used^58,65^ (see Supplementary information, Table S7 for detailed gene lists). We used a way similar to Tirosh et al.^66^ to classify the cycling phases of the cells. We calculated the average expression of each gene set as corresponding scores, and manually assigned cells to approximate cell cycle phases based on the scores. Namely, cells with G1/S score  < 2 and G2/M score < 2 were assigned as “quiescent”, otherwise “proliferative”. Among proliferative cells, those with G2/M score > G1/S score were assigned as “G2/M”, and those with G1/S score > G2/M score were assigned as “G1” when G2/M score < 2, or as “S” when G2/M score ≥ 2.

### Constructing single-cell trajectories

Monocle 2 (version 2.6.4)^67^ and Mpath (version 1.0)^68^ were adopted to infer the development trajectory of selected cell populations. Monocle 2 can construct single-cell trajectories and place each cell at its proper position in the trajectory, even a “branched” trajectory corresponding to cellular “decisions”. We followed the official vignette with recommended parameters. Briefly, UMI count data of given cell populations was used as input and genes with more than 1.5 times of fitted dispersion evaluated using dispersionTable function were identified as HVGs. To reduce the influence of cell cycle effect, HVGs not included in the direct cell cycle GO term (GO:0007049) were retained as ordering genes for the subsequent ordering cells.

For Mpath analysis, the log_2_(TPM/10 + 1) normalized data of HVGs identified by using Seurat method were used as inputs. The cluster labels defined by clustering procedures described above were used as landmark cluster assignment of individual cells. Based on the results of the Mpath analyses, we specified the starting point and developing directions according to the development time and visualized the results on t-SNE plot.

### Patterns of DEGs among multiple clusters

In the case of identification of gene patterns in more than two clusters, analysis of variance followed by Tukey’s HSD test for pairwise comparison was adopted to identify DEGs (genes with adjusted *P* value < 0.05 and fold change > 2 or < 0.5). For identification of patterns in earlyAEC, lateAEC and HEC clusters, only 1005 DEGs resulted from the pairwise comparisons of earlyAEC and lateAEC and of earlyAEC and HEC were retained. According to the changed directions of HEC and lateAEC as compared to earlyAEC, we could assign these DEGs into 8 patterns as illustrated. TF network visualization was implemented as follows. First, the average expression values of genes included in each pattern were calculated as their representative expression levels. Then, the representative expression levels of 8 patterns and the expression profile data of TFs included in these patterns were combined as input for construction of “signed hybrid” weighted gene co-expression network analysis using WGCNA.^69^ Next, we used 0.01 as adjacency threshold for including edges in the output to export network, which was then imported into Cytoscape^70^ for visualization. We also calculated Pearson correlation coefficient between each TF and the pattern it belongs to.

For identification of patterns in pEC, pAEC, earlyAEC and HEC clusters, all 2851 DEGs among them were retained. We used ConsensusClusterPlus function with k-means algorithm on top 500 DEGs to achieve five stable clusters. Then all DEGs were reassigned into one of the five patterns according to which pattern had maximum average Pearson correlation coefficient with a given DEG. Note that we used a downsampled dataset in the visualization related to the five patterns in order to show more detailed changes along the development trajectory. Sixty cells were randomly sampled from pEC and pAEC clusters, respectively.

### Identification of HEC signature genes

We first compared HEC to every cluster to get the overrepresented genes, which were up-regulated across each of the other 4 clusters (vEC, earlyAEC, lateAEC and HC) within filtered initial dataset. To make sure the accuracy of HEC overrepresented genes, we used both wilcox and roc method to perform the DEG analysis. Only the DEGs identified by both methods were regarded as HEC overrepresented genes. Finally, 25 cluster HEC-overrepresented genes were retained. In order to not only identify the endothelium with hemogenic potential, but also discriminate those HSC-primed hemogenic ECs from yolk sac-derived early hematopoietic populations such as erythro-myeloid progenitors, genes highly expressed in erythro-myeloid progenitors (*Gsta4*, *Spi1*, *Alox5ap* and *Myb*) as reported^71,72^ were excluded. In addition, genes not highly expressed (log_2_(TPM/10 + 1) < 2) in HEC or highly expressed (log_2_(TPM/10 + 1) > 2) in every clusters were also excluded. Finally, eleven HEC-overrepresented genes were retained as HEC signature genes.

### SCENIC analysis

SCENIC^73^ could reconstruct gene regulatory networks from scRNA-seq data based on co-expression and DNA motif analysis. Here, we used SCENIC R package (version 1.1.1-9) to identify refined regulons, each of which represented a regulatory network that connects a core TF with its target genes. We followed the “Running SCENIC” vignette in the R package with default settings. We identified 507 unique regulons, among which 75 regulons significantly overlapped with the 2851 significantly changed genes were retained. Fisher’s exact test was employed to estimate the statistical significance of their overlaps. The 75 core TFs were considered as putative driving force to orchestrate the dynamic molecular program during HEC specification, given the simultaneous co-expression of the core TF and its predicted targets in a given regulon.

### Gene set variation analysis

Through gene set variation analysis, gene-level expression profiles could be transformed into pathway-level enrichment score profiles using GSVA R package^74^ coupled with KEGG pathways.^75^ We used ssgsea method^76^ to estimate gene-set enrichment scores of each cell. Two-sample Wilcoxon test was employed to find differentially enriched pathways between involved clusters. Adjusted *P* value < 0.05 was considered statistically significant.

### TFs and cell surface molecules

Genes were marked as TFs according to 1485 TFs included in AnimalTFDB 2.0,^77^ and marked as surface molecules according to 871 high-confidence surfaceome proteins identified in Cell Surface Protein Atlas.^78^ See Supplementary information, Table S7 for the detailed gene lists.

### Statistical analysis

All statistical analyses were conducted in R version 3.4.3. Two-sample Wilcoxon Rank Sum test was employed for comparisons of gene numbers, transcript counts, or gene expression levels between two clusters of cells. We referred to statistically significant as *P* < 0.05 (if not specified). Network enrichment analyses and gene ontology biological process enrichment analyses were performed using Metascape (http://metascape.org)^79^ and clusterProfiler,^80^ respectively.

## Supplementary information

Supplementary Figure S1

Supplementary Figure S2

Supplementary Figure S3

Supplementary Figure S4

Table S1

Table S2

Table S3

Table S4

Table S5

Table S6

Table S7

## Data Availability

The scRNA-seq data have been deposited in the NCBI Gene Expression Omnibus under accession number GSE139389.

## Code availability

Code is available on reasonable request.
