# Supplementary material for: Embryonic endothelial evolution towards first hematopoietic stem cells revealed by single-cell transcriptomic and functional analyses
Source: Cell Res. 2020 Mar 20;30(5):376–92. doi: 10.1038/s41422-020-0300-2 (PMC7196075; doi:10.1038/s41422-020-0300-2)
Supplement: Supplementary file 1 — Supplementary Figure S1 [file 41422_2020_300_MOESM1_ESM.pdf]

Fig. S1

a

Stage	Embryos	Exp. (n)	Body	DA	Neg[a]	Total
E9.5 (25 sp)	4	2[b]	180	/	40	220
E10.0 (31-35 sp)	10	2[b]+2[c]	180	110	40	330
E10.5 (37-39 sp)	8	1[c]	/	56	/	56
E 11.0 (43-44 sp)	7	1[c]	/	56	/	56
Total	29	8	360	222	80	662

[a] $CD45^-CD31^-CD144^-$ negative control cells from body;
[b] independent experiments for collecting $CD45^-CD31^+CD144^+$ sample cells from body;
[c] independent experiments for collecting $CD45^-CD31^+CD144^+$ Oregon green$^+$ sample cells from AGM region.

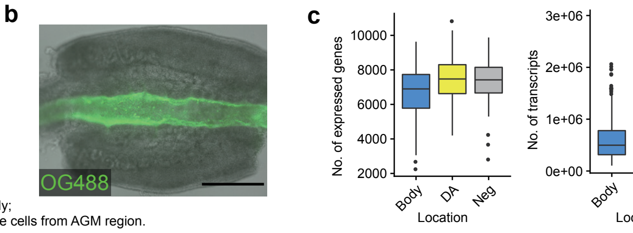

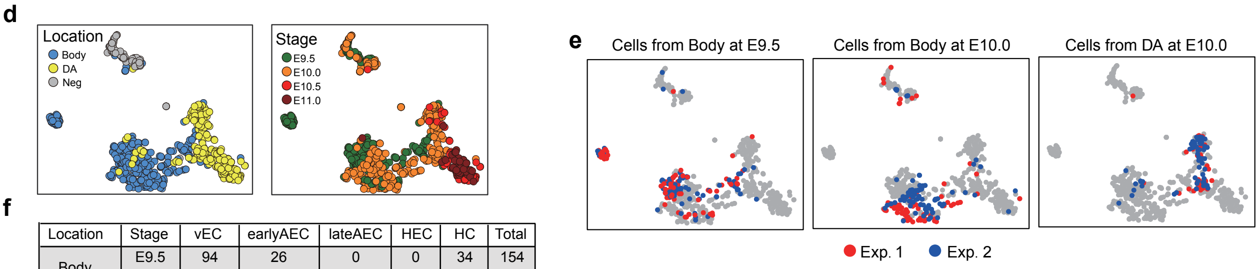

f

Location	Stage	vEC	earlyAEC	lateAEC	HEC	HC	Total
Body	E9.5	94	26	0	0	34	154
	E10.0	118	14	1	2	0	135
DA	E10.0	10	42	0	33	0	85
	E10.5	0	4	35	4	0	43
	E11.0	1	2	43	0	0	46
Total		223	88	79	39	34	463

Cells excluding Neg cluster, *Ptprc*- or *Spn*-expressing cells

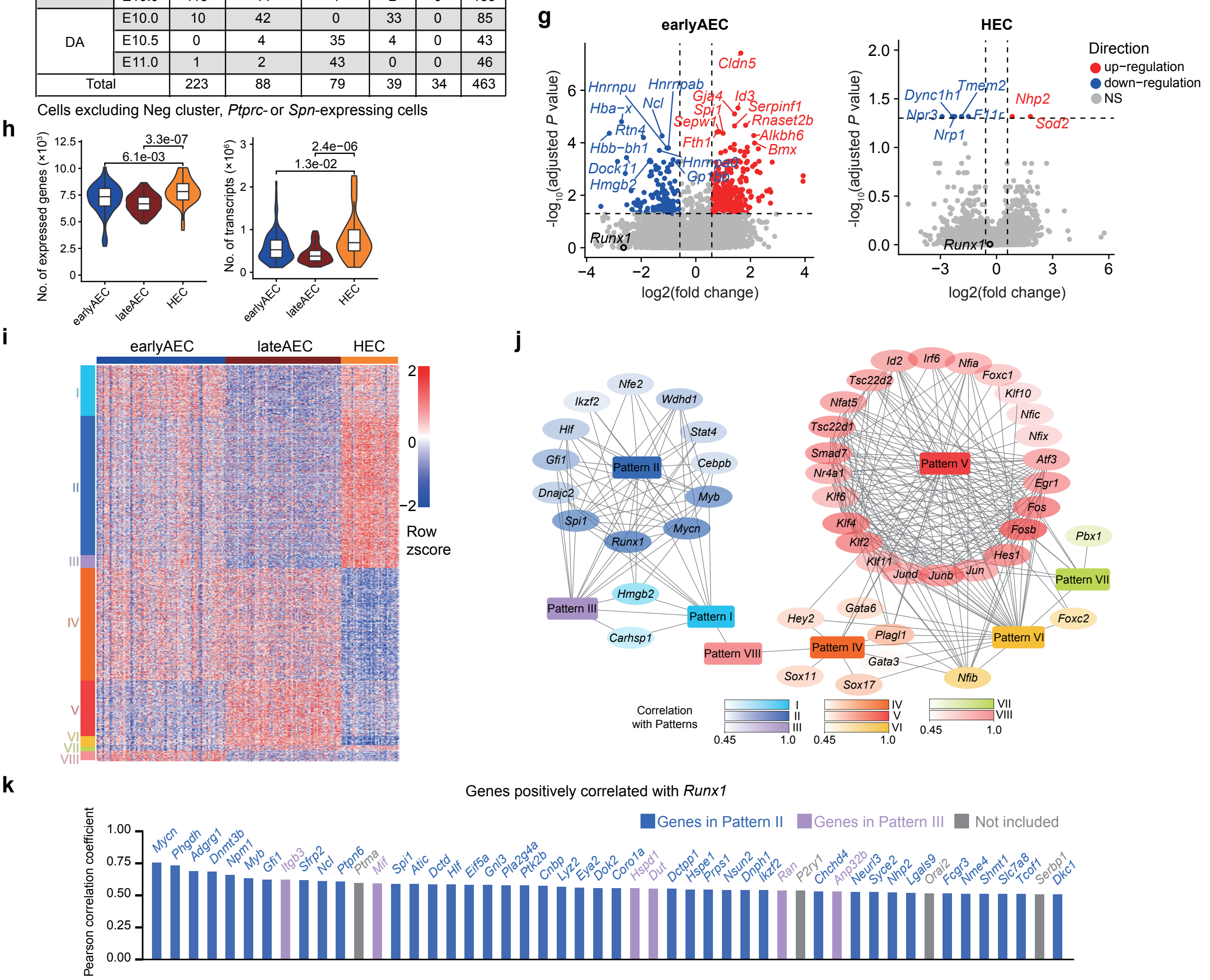

Supplementary Figure 1. Information, clustering of initial dataset and molecular characteristics of major clusters. **a.** Embryo, independent experiment, and cell number information for scRNA-seq. DA, dorsal aortic luminal layer of AGM region. **b.** Whole-mount image of the E11.0 AGM region labeled with Oregon Green 488. Scale bar, 500 μm. **c.** Boxplots showing the number of genes (left) and transcripts (right) in each single cell of different locations. **d.** t-SNE plots with sampling locations (right) and embryonic stages (right) mapped onto it. **e.** t-SNE plots with cells sampled from different experiments (Exp.) mapped onto it. Note no batch effect was observed. **f.** Cell number information of the spatiotemporal distribution of distinct clusters. **g.** Volcano plots showing differentially expressed genes (marked as blue or red) between two sub-clusters by forced clustering in earlyAEC and HEC, respectively. Top 10 (earlyAEC) or all (HEC) differentially expressed genes are indicated. Runx1 is also indicated. **h.** Violin plots showing the number of genes (left) and transcripts (right) in each single cell of the indicated clusters. Wilcoxon Rank Sum test is employed to test the significance of difference and *P* values are indicated for the comparison. $P < 0.05$ is considered statistically significant. **i.** Heatmap showing the relative expression levels of genes in eight patterns among earlyAEC, lateAEC and HEC. **j.** Network view of TFs positively correlated with the gene expression patterns. A deeper background color of the gene name indicates a higher positive correlation of the TF to that expression pattern. **k.** Bar chart showing the top 50 genes positively correlated with *Runx1* within cell population including earlyAEC, lateAEC and HEC. Genes included in the patterns identified above are marked as indicated.
