# Supplementary material for: Embryonic endothelial evolution towards first hematopoietic stem cells revealed by single-cell transcriptomic and functional analyses
Source: Cell Res. 2020 Mar 20;30(5):376–92. doi: 10.1038/s41422-020-0300-2 (PMC7196075; doi:10.1038/s41422-020-0300-2)
Supplement: Supplementary file 2 — Supplementary Figure S2 [file 41422_2020_300_MOESM2_ESM.pdf]

Fig. S2

a

Stage	Region	Exp (n)	Population	Constitution in the region (%)	Cell dose[a]	Clusters[b]
E9.5-E10.0 (30-32 sp[c])	Caudal half	4	CD41$^-$CD43$^-$CD45$^-$CD31$^+$Kit$^+$CD201$^+$	0.20±0.07	3.0-4.0 ee[d]	13/13 (100%)
			CD41$^-$CD43$^-$CD45$^-$CD31$^+$Kit$^+$CD201$^-$	1.25±0.45	3.0-4.0 ee	2/13 (15.4%)
E9.5-E10.0 (30-34 sp)	Caudal half	4	CD41$^-$CD43$^-$CD45$^-$CD201$^+$CD44$^+$	0.09±0.03	3.0-4.0 ee	11/11 (100%)
			CD41$^-$CD43$^-$CD45$^-$CD201$^+$CD44$^-$	0.33±0.21	3.0-4.0 ee	0/11

[a]cell dose per well and per recipient. [b]hematopoietic cluster positive wells/total wells. [c]somite pairs. [d]embryo equivalents.

b

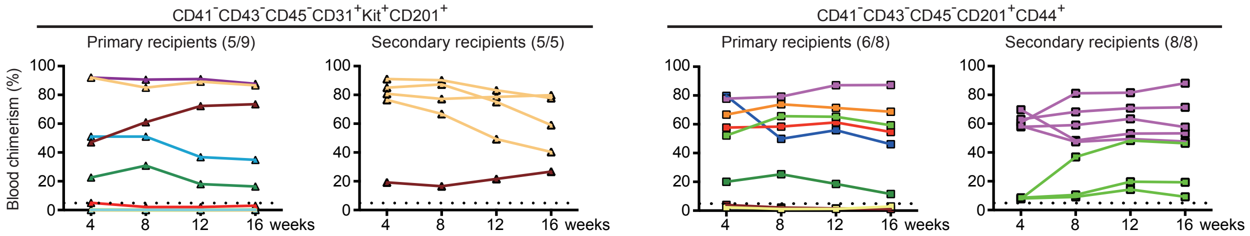

c

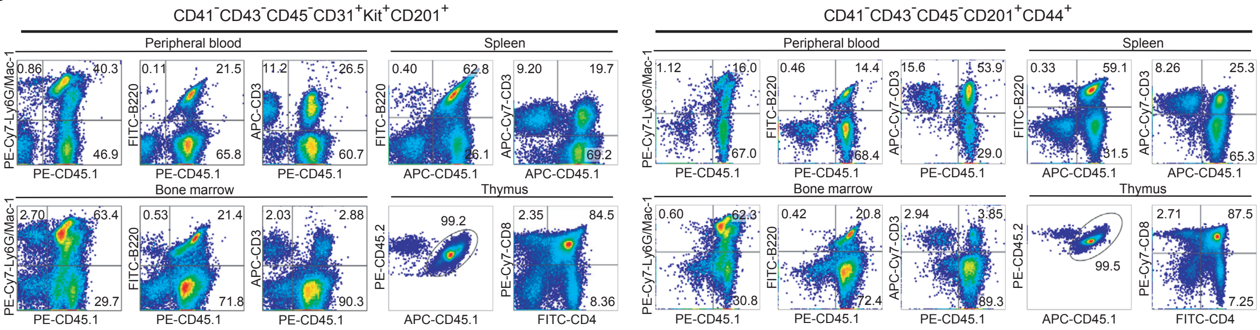

d

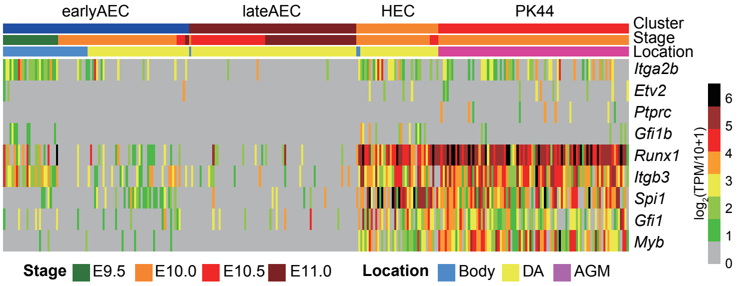

e

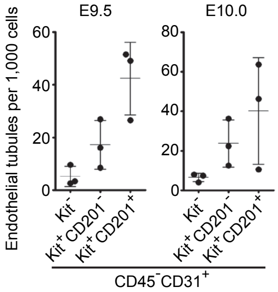

f

Stage	Region	Exp (n)	Population in EC (CD41$^-$CD43$^-$CD45$^-$CD31$^+$)	Constitution in the region (%)	Cell dose[a]	Endothelial only[b]	Endothelial and hematopoietic[c]	Hematopoietic only[d]
E9.5-E10.0 (29-31 sp[e])	Caudal half	2[f]	Kit$^+$CD201$^+$CD44$^+$	0.11±0.03	10 cells	0/44	35/44	9/44
			Kit$^+$CD201$^+$CD44$^-$	0.10±0.02	10 cells	33/42	0/42	0/42
E9.5-E10.0 (30-32 sp)	AGM	5[g]	Kit$^+$CD201$^+$CD44$^+$	0.31±0.09	Single cell	60/261 (1:4)	7/261 (1:37)	106/261 (1:2)

[a]number of cells per well. [b]only endothelial tube positive wells/total wells. [c]both endothelial and hematopoietic progeny positive wells/total wells.
[d]only hematopoietic progeny positive wells/total wells. [e]somite pairs. [f]2 independent experiments, totally 12 embryos were used.
[g]5 independent experiments, totally 15 embryos were used.

Supplementary Figure 2. Identification of the HSC-competent and endothelial-hematopoietic dual-potent HECs. **a.** Detailed information of the co-culture/transplantation assays performed with E9.5-E10.0 caudal half cells. **b.** Blood chimerism of the primary and secondary recipients at 4-16 weeks post-transplantation. The primary recipients were transplanted with the derivatives of the indicated cell populations from the caudal half of E9.5-E10.0 embryos. The paired primary and corresponding secondary repopulated mice are show as the same symbol and color. Numbers of repopulated/total recipients are shown in the brackets. Only the recipients survived to 16 weeks post-transplantation are shown. **c.** FACS plots showing representative primary recipients with long-term (16 weeks), multi-organ and multi-lineage repopulations transplanted with the derivatives of the indicated cell populations from the caudal half of E9.5-E10.0 embryos. Donor-derived ($CD45.1^+CD45.2^+$) myeloid ($Gr\text{-}1^+/Mac\text{-}1^+$), B lymphoid ($B220^+$), and T lymphoid ($CD3^+$) cells in multiple hematopoietic organs are shown. **d.** Heatmap showing the expression of selected genes in earlyAEC, lateAEC, HEC and PK44 populations. Note the similarity of expression patterns between HEC and PK44. **e.** Graph showing the endothelial potential of different cell populations in E9.5-E10.0 body part of embryo proper. Cells with indicated immunophenotype were isolated by FACS, co-cultured with OP9 stromal cells for 7 days, and stained with CD31 to identify the endothelial tubes. Data are means ± s.d.. For E9.5 embryos, data are from 3 independent experiments with 6-9 embryo equivalents pooled for each experiment. For E10.0 embryos, data are from 3 independent experiments with 8-9 embryo equivalents pooled for each experiment. **f.** Detailed information of endothelial-hematopoietic dual-potential induction assays performed with cells from E9.5-E10.0 caudal half or AGM region.
