# Supplementary material for: Embryonic endothelial evolution towards first hematopoietic stem cells revealed by single-cell transcriptomic and functional analyses
Source: Cell Res. 2020 Mar 20;30(5):376–92. doi: 10.1038/s41422-020-0300-2 (PMC7196075; doi:10.1038/s41422-020-0300-2)
Supplement: Supplementary file 3 — Supplementary Figure S3 [file 41422_2020_300_MOESM3_ESM.pdf]

Fig. S3

a

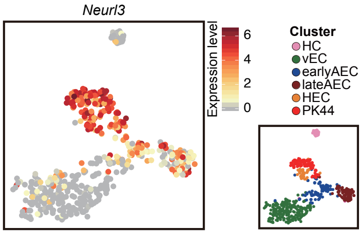

b

Stage	Region	Exp (n)	Population in EC ($CD41^-CD43^-CD45^-CD31^+$)	Cell dose[a]	Clusters[b]
E9.5-E10.0 (30-34 sp[c])	Caudal half	5	$CD44^+$Neurl3-$EGFP^+$	1.5-2.5 ee[d]	6/6 (100%)
			$CD44^+$Neurl3-$EGFP^-$	1.5-5.0 ee	3/6 (50%)

[a]cell dose per recipient. [b]hematopoietic cluster positive wells/total wells. [c]somite pairs.
[d]embryo equivalents.

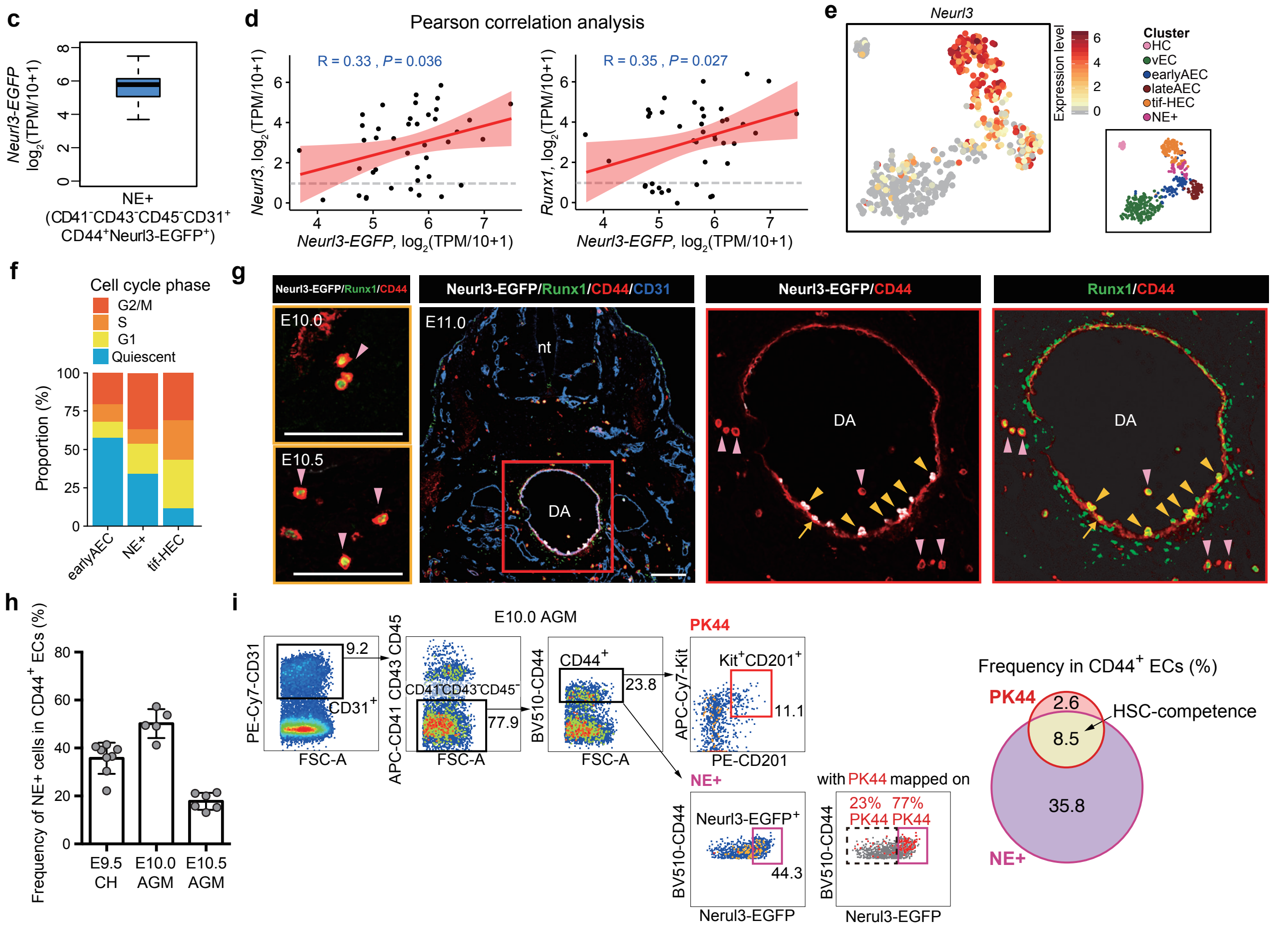

j

Single-cell co-culture

Stage	Region	Exp (n)	Population in EC ($CD41^-CD43^-CD45^-CD31^+$)	Constitution in the region (%)	Constitution in EC (%)	Constitution in NE+[a] (%)	Endothelial only[b]	Endothelial and hematopoietic[c]	Hematopoietic only[d]
E9.5 (26-30 sp[e])	Caudal half	8[f]	$CD44^+$Neurl3-$EGFP^+$	0.20±0.07	2.70±0.66	100.0	42/301 (1:7)	1/301 (1:301)	25/301 (1:12)
					PK44 within NE+	39.5	28/119 (1:4)	1/119 (1:119)	17/119 (1:7)
E10.0 (31-35 sp)	AGM	5[g]	$CD44^+$Neurl3-$EGFP^+$	1.20±0.57	11.3±3.51	100.0	49/467 (1:10)	2/467 (1:234)	37/467 (1:13)
					PK44 within NE+	29.3	21/137 (1:7)	2/137 (1:69)	32/137 (1:4)
E10.5 (36-39 sp)	AGM	6[h]	$CD44^+$Neurl3-$EGFP^+$	0.29±0.10	2.74±0.52	100.0	32/159 (1:5)	1/159 (1:159)	5/159 (1:32)
					PK44 within NE+	51.6	20/82 (1:4)	1/82 (1:82)	5/82 (1:16)

[a]NE+: $CD41^-CD43^-CD45^-CD31^+CD44^+$Neurl3-$EGFP^+$ cells. [b]only endothelial tube positive wells/total wells. [c]both endothelial and hematopoietic progeny positive wells/total wells.
[d]only haematopoietic progeny positive wells/total wells. [e]somite pairs. [f]8 independent expreiments, totally 32 embryos were used.
[g]5 independent expreiments, totally 37 embryos were used. [h]6 independent expreiments, totally 23 embryos were used.

Supplementary Figure 3. Identification of the HSC-competent HECs marked by Neurl3-EGFP reporter. **a.** Expression of *Neurl3* exhibited on t-SNE plot. The same t-SNE plot with the clusters of interest (HC, vEC, earlyAEC, lateAEC, HEC, and PK44) mapped on it is shown to the right. **b.** Detailed information of the co-culture/transplantation assays performed with the caudal half cells from E9.5-E10.0 *Neurl3*$^{EGFP/+}$ embryos. **c.** Boxplot showing the transcriptional expression level of *EGFP* in NE+ cell population, NE+, $CD41^-CD43^-CD45^-CD31^+CD44^+$Neurl3-$EGFP^+$ population from AGM region of E10.0 *Neurl3*$^{EGFP/+}$ embryos. **d.** Scatter plots showing correlation of the expression of *EGFP* with that of *Neurl3* and *Runx1*, respectively. Fitted line and 95% confidence interval are shown in red. Pearson correlation coefficients and *P* values are also shown in blue text. **e.** Expression of *Neurl3* exhibited on t-SNE plot. The same t-SNE plot with the clusters of interest (HC, vEC, earlyAEC, lateAEC, tif-HEC, and NE+) mapped on it is shown to the right. **f.** Stacked bar chart showing the constitution of different cell cycle phases in the indicated clusters. **g.** Representative immunostaining on cross sections at AGM region of E10.0-E10.5 (left column) and E11.0 (right three panels) *Neurl3*$^{EGFP/+}$ embryos. Arrow indicates Neurl3-EGFP+ aortic ECs; Yellow arrowheads indicate Neurl3-EGFP+ bulging and bulged cells and also IAHCs. White pink arrowheads indicate $CD44^+Runx1^+$Neurl3-$EGFP^-$ hematopoietic cells distributed outside the aorta. Images in yellow boxes show the corresponding high magnification views of those in Fig. 5a. nt, neural tube; DA, dorsal aorta. Scale bars, 100 µm. **h.** Graph showing the frequencies of the NE+ ($CD41^-CD43^-CD45^-CD31^+CD44^+$Neurl3-$EGFP^+$) cells in $CD44^+$ ECs in E9.5 caudal half (CH) or E10.0-E10.5 AGM region of *Neurl3*$^{EGFP/+}$ embryos. Data are means ± s.d.. Data are from 8, 5, and 6 independent experiments for E9.5, E10.0, and E10.5 embryos, respectively. **i.** Representative FACS plots and the corresponding diagram to the right showing the relationship between PK44 and NE+ populations in E10.0 AGM of *Neurl3*$^{EGFP/+}$ embryos. **j.** Detailed information of endothelial-hematopoietic dual-potential induction assays performed with cells from E9.5 caudal half and E10.0-E10.5 AGM region of *Neurl3*$^{EGFP/+}$ embryos.
