# Supplementary material for: Embryonic endothelial evolution towards first hematopoietic stem cells revealed by single-cell transcriptomic and functional analyses
Source: Cell Res. 2020 Mar 20;30(5):376–92. doi: 10.1038/s41422-020-0300-2 (PMC7196075; doi:10.1038/s41422-020-0300-2)
Supplement: Supplementary file 4 — Supplementary Figure S4 [file 41422_2020_300_MOESM4_ESM.pdf]

Fig. S4

a

Stage	Embryos	Exp. (n)	Sample[a]	Neg[b]	Total
E8.0 (3-5 sp)	4	1	40	8	48
E8.5 (8-9 sp)	14	3	236	50	286
E9.0 (17-18 sp)	6	2	205	40	245
Total	24	6	481	98	579

[a]CD45⁻CD31⁺CD144⁺ sample cells from body
[b]CD45⁻CD31⁻CD144⁻ negative control cells from body

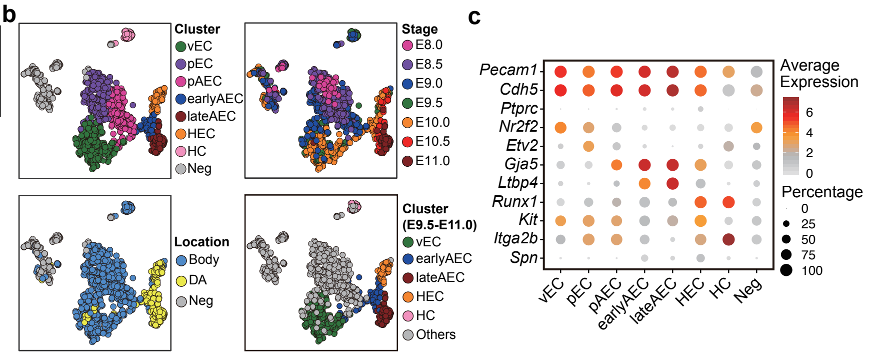

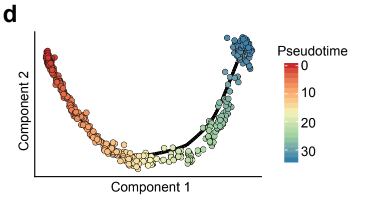

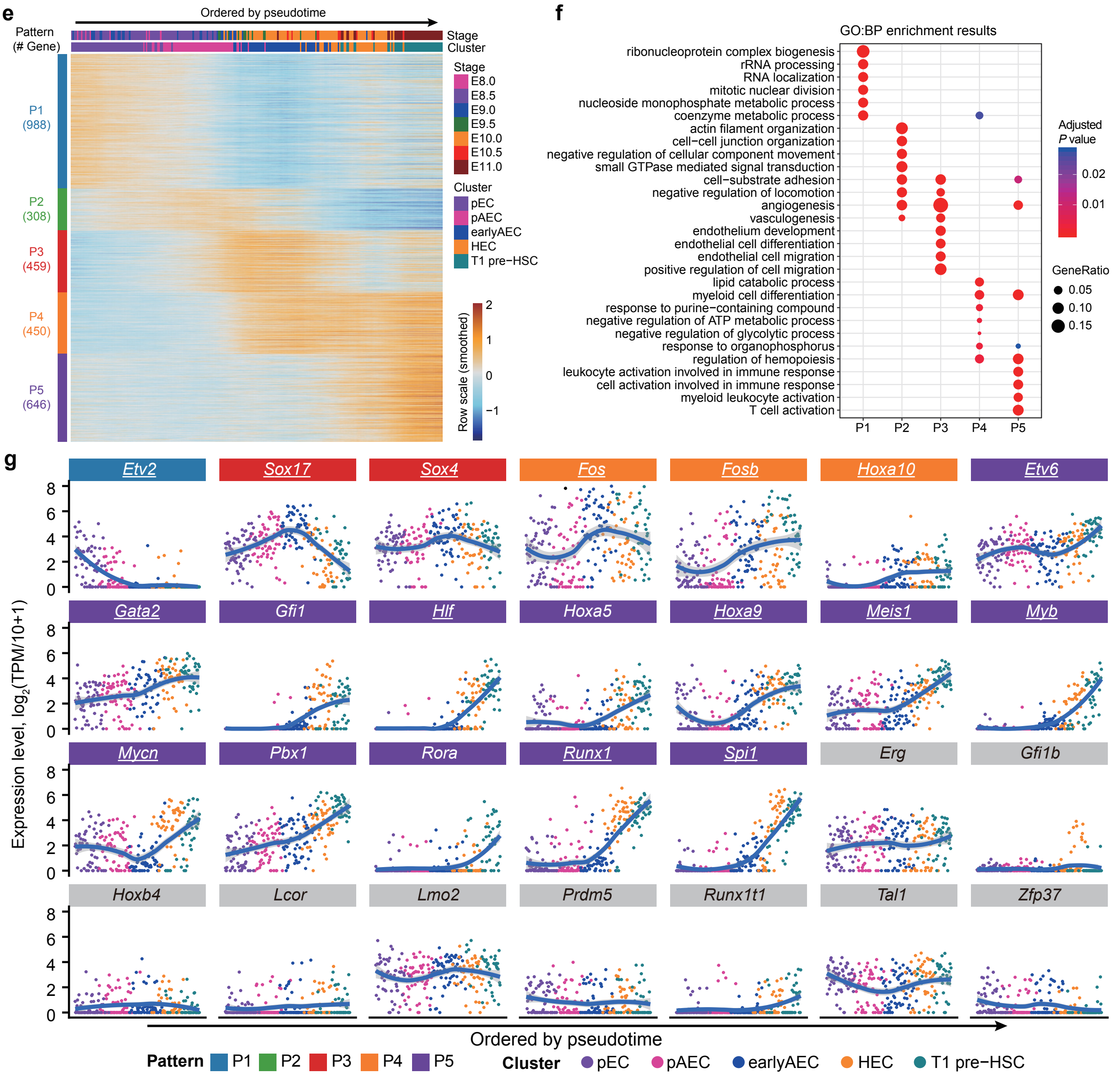

Supplementary Figure 4. Molecular programs from primitive vascular ECs to HSC-primed HECs. **a.** Embryo, independent experiment, and cell number information for additional scRNA-seq. sp, somite pairs. **b.** t-SNE plots with clusters (upper left), sampling locations (lower left), embryonic stages (upper right) and clusters previously defined (lower right) mapped onto it. **c.** Dot plot showing the average and percentage expression of selected marker genes in the indicated clusters. **d.** Pseudotemporal ordering of the cells involved in HEC specification, including those in pEC, pAEC, earlyAEC, HEC, and T1 pre-HSC, inferred by monocle 2, with pseudotime mapped to it. **e.** Heatmap showing smoothed and scaled expression levels of 2 851 pattern genes. Genes are ordered by patterns. Cells are ordered by pseudotime. **f.** Dot plot showing the top six enriched Gene Ontology biological process (GO:BP) terms for each pattern. Dot color indicates statistical significance of the enrichment and dot size represents the fraction of genes annotated to each term. **g.** Scatter plots showing the expression levels of the TF genes previously reported to be functional in HSPC regeneration along the pseudotemporal order with loess smoothed fit curves and 95% confidence interval indicated. The patterns to which the genes belong are indicated by different fill colors. The core TFs of the significantly overlapped regulons are underlined.
